# Serum Amyloid A in Inflammatory Rheumatic Diseases: A Compendious Review of a Renowned Biomarker

**DOI:** 10.3389/fimmu.2020.631299

**Published:** 2021-02-19

**Authors:** Iva Sorić Hosman, Ivanka Kos, Lovro Lamot

**Affiliations:** ^1^ Department of Pediatrics, Zadar General Hospital, Zadar, Croatia; ^2^ Division of Nephrology, Dialysis and Transplantation, Department of Pediatrics, University Hospital Centre Zagreb, Zagreb, Croatia; ^3^ Department of Pediatrics, University of Zagreb School of Medicine, Zagreb, Croatia

**Keywords:** serum amyloid A, biomarkers, markers of inflammation, rheumatic inflammatory disease, autoinflammatory disease, amyloidosis, biological therapy, COVID-19

## Abstract

Serum amyloid A (SAA) is an acute phase protein with a significant importance for patients with inflammatory rheumatic diseases (IRD). The central role of SAA in pathogenesis of IRD has been confirmed by recent discoveries, including its involvement in the activation of the inflammasome cascade and recruitment of interleukin 17 producing T helper cells. Clinical utility of SAA in IRD was originally evaluated nearly half a century ago. From the first findings, it was clear that SAA could be used for evaluating disease severity and monitoring disease activity in patients with rheumatoid arthritis and secondary amyloidosis. However, cost-effective and more easily applicable markers, such as C-reactive protein (CRP) and erythrocyte sedimentation rate (ESR), overwhelmed its use in clinical practice. In the light of emerging evidences, SAA has been discerned as a more sensitive biomarker in a wide spectrum of IRD, especially in case of subclinical inflammation. Furthermore, a growing number of studies are confirming the advantages of SAA over many other biomarkers in predicting and monitoring response to biological immunotherapy in IRD patients. Arising scientific discoveries regarding the role of SAA, as well as delineating SAA and its isoforms as the most sensitive biomarkers in various IRD by recently developing proteomic techniques are encouraging the revival of its clinical use. Finally, the most recent findings have shown that SAA is a biomarker of severe Coronavirus disease 2019 (COVID-19). The aim of this review is to discuss the SAA-involving immune system network with emphasis on mechanisms relevant for IRD, as well as usefulness of SAA as a biomarker in various IRD. Therefore, over a hundred original papers were collected through an extensive PubMed and Scopus databases search. These recently arising insights will hopefully lead to a better management of IRD patients and might even inspire the development of new therapeutic strategies with SAA as a target.

## Introduction

Serum amyloid A (SAA) is the most prominent acute phase reactant as its serum levels in acute phase response demonstrate the most notable increase. In healthy individuals, SAA is present at the blood concentration below 3 mg/L. During the acute phase of the inflammatory response, SAA increases up to 1,000-fold in 24 h by stimulation of the pro-inflammatory cytokines. This effect is followed by a rapid decline which implies a remarkable feedback regulation (1). SAA shares many similarities with the C-reactive protein (CRP), the most commonly used serum biomarker for assessing disease severity in inflammatory rheumatic diseases (IRD). Both SAA and CRP concentration increases rapidly following an inflammatory stimuli, mostly as a result of the increased synthesis in hepatocytes (1, 2). Moreover, they share some intracellular signaling pathways. In fact, both are induced by interleukin (IL)-6 and addition of IL-1 to IL-6 has a synergistic effect on their synthesis (3). Serum levels of SAA and CRP show a close relationship and are usually significantly positively correlated with each other in a wide range of clinical conditions (4). SAA is significantly elevated in patients with IRD and is commonly used for evaluating disease severity and monitoring disease activity (5). However, the non-superiority of SAA to the other commercially available inflammatory markers, such as CRP and the erythrocyte sedimentation rate (ESR), as well as technical difficulties in measuring the SAA levels have led to neglection of SAA in everyday clinical practice of not IRD specialized centres.

Lately, arising scientific discoveries regarding the role of SAA in IRD as well as the development of proteomic techniques for serum biomarker analysis encouraged a revival of clinical use of SAA. There are more than few arguments in favor of using SAA over CRP in several clinical scenarios associated with inflammation. Firstly, increased concentrations of SAA despite normal levels of CRP and ESR are frequently observed in IRD patients with mild disease activity, while increased CRP or ESR levels with normal SAA concentration are extremely rarely observed. Moreover, the low-grade inflammation with persistent elevated SAA values is associated with the development of life-threatening complications – secondary amyloidosis and coronary heart disease (6, 7). Furthermore, unlike CRP, SAA is locally expressed in inflamed synovial tissue and is directly involved in the pathogenesis of IRD by multiple immunomodulatory and cytokine-like properties, making SAA a potential therapeutic target. Therefore, it is not surprising that accumulating evidences suggests SAA as a more reliable biomarker than CRP or ESR for monitoring disease activity in various rheumatic and autoinflammatory diseases, including rheumatoid arthritis (RA), ankylosing spondylitis (AS), juvenile idiopathic arthritis (JIA), systemic lupus erythematosus (SLE), different types of vasculitis, sarcoidosis, familial Mediterranean fever (FMF), secondary amyloidosis, etc., especially in the era of biologic immunosuppressive therapy.

The aim of this review is to give a brief insight into the complex network of the multiple SAA roles in the pathogenesis of inflammation, as well as to summarize and discuss the current evidences of its clinical utility in assessing an early diagnosis and monitoring the disease activity and response to therapy in a wide range of IRD.

## Literature Review

**Figure 1:**
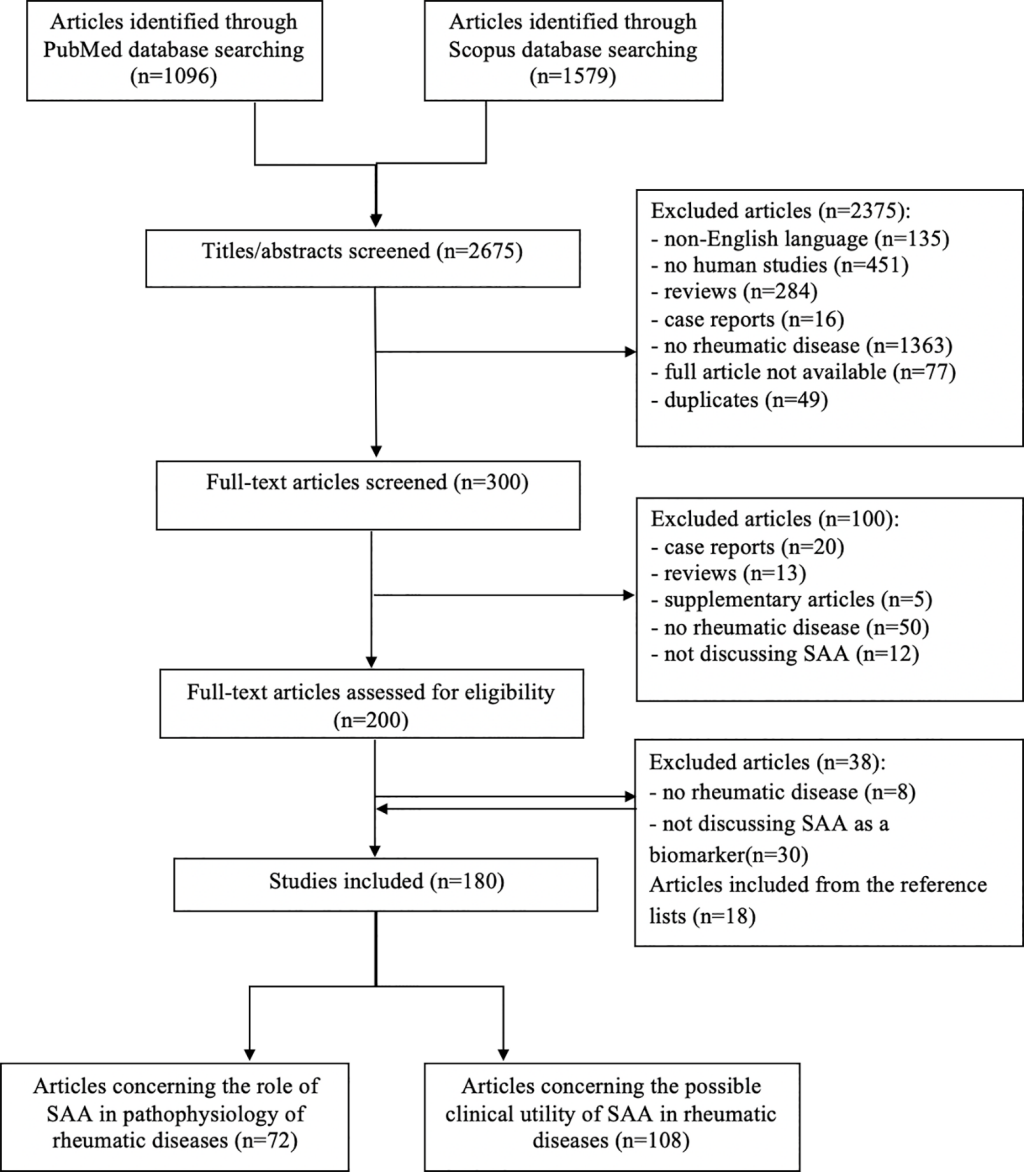
Flow chart illustrating the literature search and study selection process.

A comprehensive literature review was conducted using PubMed and Scopus databases to identify articles exploring the role and utility of SAA in IRD, according to the published guidance on narrative reviews (8). We used search terms of “serum amyloid A”, “serum biomarkers”, “markers of inflammation”, “rheumatic disease”, “autoinflammatory disease” and “COVID-19” in different combinations. The latter term was added since we are currently experiencing a pandemic of Coronavirus disease 2019 (COVID-19). Search terms were used as key words and as MeSH terms to maximize the output from the literature. Only available full-text articles in English published until September 2020 were included. Additional exclusion criteria were studies on non-human species, case reports, reviews, commentaries and studies not concerning rheumatic diseases or not discussing SAA. The reference lists of the selected articles were reviewed to identify additional articles meeting the eligibility criteria. The database search resulted in 2675 articles of which 300 remained after the removal of duplicates and title/abstract screening. Finally, after assessing the full-text articles for eligibility and screening of the reference lists, a total of 180 full-text articles were included in the present review. The included articles were divided in two groups: articles reporting on the role of the SAA-related genes and proteins in the pathogenesis of IRD (n=72) and articles reporting on correlations between SAA and clinical features of various IRD (n=102) or COVID-19 (n=6) (**Figure 1**). Results from the first group are summarized in the “SAA-related genes and proteins” and “SAA in rheumatic diseases” sections, while results from the latter group are summarized in a narrative manner in each relevant section of this review. Summary tables with characteristics of each article included in each section are provided (**Tables 1** **–** **9**).

**Table 1:** Characteristics and results of articles investigating clinical utility of SAA in patients with rheumatoid arthritis.

First author (reference number)	The aim of the study regarding the clinical utility of SAA	Number of patients	Diagnosis(number of patients)	Method used for SAA measuring	Results / Comments
Benson and Cohen (5)	To investigate the possible uses of SAA in rheumatic diseases	277	RA (n=65) JIA (n=26) SLE (n=25) OA (n=23) HC (n=138)	Radioimmuno- assay	➢ SAA is significantly elevated in RA, JIA and SLE patients compared to HC and patients with degenerative joint disease ➢ SAA is significantly correlated with ESR and with disease activity in RA
De Beer et al. (9)	To evaluate SAA as a marker of diagnosis and severity in rheumatic diseases	266	RA (n=99) JIA (n=24) SLE (n=43) HC (n=100)	Radioimmuno- assay	➢ SAA is significantly elevated in RA, JIA and SLE patients compared to HC ➢ SAA is a marker of disease activity in RA and JIA, but is not significantly correlated with disease activity in SLE patients
Maury et al. (10)	To evaluate SAA and CRP levels in RA and SLE patients	65	RA (n=48) SLE (n=17)	Radioimmuno-assay	➢ SAA is significantly correlated with disease activity in RA patients, but not in SLE patients ➢ SAA levels are strongly correlated with CRP levels in RA and SLE patients
Shen et al. (11)	To evaluate possible uses of SAA in patients with RA	265	RA (n=88) OA (n=54) SLE (n=43) Other AID (n=30) HC (n=50)	ELISA and western blot analysis	➢ SAA can be used as a marker for diagnosis of RA (among patients with other autoimmune diseases or osteoarthritis) ➢ SAA significantly correlates with RA disease activity (measured as DAS28-ESR)
Kumon et al. (12)	To compare SAA levels in serum and synovial fluid between OA and RA patients	55	RA (n=34) OA (n=21)	ELISA	➢ SAA levels are significantly higher in both serum and synovial fluid in RA patients compared to OA patients and therefore can be used as a marker for RA diagnosis
Targonska-Stepniak et al. (13)	To assess SAA level in RA patients and its correlation with cardiovascular and renal involvement	140	RA (98 high activity + 42 low activity)	ELISA	➢ SAA is a sensitive marker of RA and is significantly correlated with disease activity ➢ SAA is an indicator of cardiovascular and renal involvement in RA patients
Chambers et al. (14)	To assess SAA as a marker of disease activity in RA patients	385	RA (n=185) HC (n=200)	Radioimmuno-assay	➢ SAA is a more sensitive marker of disease activity in RA than CRP
Cunnane et al. (15)	To compare SAA with CRP and ESR in relation to diagnosis and disease activity in early inflammatory arthritis	140	RA (n=64) PsA (n=19) UA (n=57)	ELISA	➢ Compared with CRP and ESR, SAA correlates best with disease activity ➢ SAA, unlike CRP or ESR, can be used for distinguishing patients with a final diagnosis of RA in early inflammatory arthritis
Yoo et al. (16)	To evaluate serum and exosomal AA levels in RA patients	60	RA (30 with active disease + 30 with inactive disease)	ELISA	➢ both serum and exosomal AA may be used as a RA disease activity biomarker
Ostensen et al. (17)	To evaluate SAA as a marker of disease activity during pregnancy in RA and AS patients	52	RA (n=11) AS (n=13) HC (n=28)	Radioimmuno-assay	➢ SAA is a reliable marker of disease activity in RA and AS even during pregnancy
Hwang et al. (18)	To evaluate use of SAA in monitoring disease activity and therapy response in RA patients	594	RA	Immunonephelo-metric assay	➢ SAA correlates better than CRP with RA disease activity, especially during treatment with TNFα antagonists
Wild et al. (19)	To compare the utility of different biomarkers as diagnostic indicators of RA	645	RA (n=364) OA (n=281)	ELISA	➢ SAA is the only biomarker (among 131 initially considered in the study) that increases sensitivity of anti-CCP for RA diagnosis ➢ sensitivity and specificity of SAA are higher than those of CRP for diagnosing RA
De Seny et al. (20)	To compare SAA levels in serum and synovial fluid between OA and RA patients	91	RA (n=27) OA (n=29) HC (n=35)	ELISA	➢ SAA levels are significantly higher in both serum and synovial fluid in RA patients compared to OA patients and healthy controls and therefore can be used as a marker for RA diagnosis
Ally et al. (21)	To evaluate serum biomarkers as markers of disease activity in early RA	128	RA	Immunonephelo-metric assay	➢ SAA is significantly correlated with disease activity in DMARD-naive patients with early RA
Targonska-Stepniak et al. (22)	To investigate effects of leflunomide therapy on SAA concentrations and disease activity in RA patients	50	RA (13 with SAA<50 mg/L + 37 with SAA> 50mg/L)	ELISA	➢ Baseline SAA can be used for predicting response to leflunomide (cut off value 50 mg/L) ➢ In spite of a significant reduction in ESR and CRP in RA patients receiving leflunomide, high SAA levels may persist and can be used for detecting subclinical inflammation and adjusting treatment
Connolly et al. (23)	To investigate the relationship between SAA and disease progression in RA and PsA patients undergoing biologic therapy	62	RA (n=45) PsA (n=17)	ELISA	➢ SAA significantly correlates with RA and PsA disease activity ➢ SAA is independently associated with 1-year radiographic progression in RA ➢ SAA is a more accurate predictor of radiographic progression and a more sensitive biomarker of disease activity in patients receiving biologic therapy compared with CRP or ESR
Centola et al. (24)	To develop a multi-biomarker disease activity (MBDA) test for RA	702	RA	ND	➢ SAA is a marker of disease activity incorporated in MBDA test
Ma et al. (25)	To explore the utility of serum biomarkers incorporated in MBDA for predicting remission in RA patients	148	RA	ELISA	➢ baseline SAA levels are significantly correlated with remission at 1 year in RA patients ➢ SAA levels are significantly higher in RA patients with low disease activity compared to those in remission and therefore can be used for detecting minimal inflammation
Wong et al. (26)	To relate serum biomarkers to vascular elasticity in RA patients	106	RA (n=53: 15 with + 38 without coronary artery disease) HC (n=53)	Immunonephelo-metric assay	➢ SAA levels are significantly inversely correlated with arterial elasticity in RA patients, indicating cardiovascular disease
Rho et al. (27)	To evaluate SAA as a marker of atherosclerosis severity in RA patients	261	RA (n=169) HC (n=92)	ELISA	➢ SAA shows a trend of association with the severity of coronary atherosclerosis in RA patients
Kullich et al. (28)	To evaluate effect of leflunomide on serum biomarker levels	36	RA	ELISA	➢ SAA is a sensitive marker of response to leflunomide (DMARD) therapy in RA patients
Momohara et al. (29)	To evaluate SAA as a marker of disease activity in RA	140	RA	ELISA	➢ SAA is a more sensitive marker of RA disease activity than CRP or ESR ➢ SAA is highly expressed in chondrocytes from RA patients
Boeters et al. (30)	To identify biomarkers for predicting sustained DMARD-free remission in RA patients	299	RA	ELISA	➢ SAA level at disease onset may be used as a predictor of achieving DMARD-free remission in ACPA-negative RA patients
Migita et al. (31)	To explore effects of tofacitinib on inflammatory biomarkers in RA patients	14	RA	Immunonephelo-metric assay	➢ SAA is a sensitive indicator of response to tofacitinib in RA patients
Visvanathan et al. (32)	To evaluate the effect of golimumab on SAA levels in RA patients	137	RA	ELISA	➢ SAA may be used for monitoring response to golimumab in RA patients ➢ Measuring SAA level at week 4 after initiation of golimumab therapy can be used to predict clinical response at week 16
Doyle et al. (33)	To investigate effects of golimumab on inflammatory biomarkers in RA patients	49	RA	ND	➢ SAA is a sensitive biomarker of response to golimumab in RA patients
Kobayashi et al. (34)	To analyze effects of adalimumab on inflammatory biomarkers in RA patients	20	RA	2DE + MS	➢ SAA is a sensitive biomarker for response to adalimumab in RA patients
Berner Hammer et al. (35)	To examine effects of adalimumab on inflammatory biomarkers in RA patients	20	RA	Immunonephelo-metric assay	➢ SAA is a sensitive biomarker for monitoring response to adalimumab in RA patients
Gabay et al. (36)	To evaluate effects of adalimumab and sarilumab on inflammatory biomarkers in RA patients	307	RA	ND	➢ SAA is a marker of disease activity in RA ➢ SAA is a sensitive biomarker of response to both anti-IL-6 and anti-TNFα therapy in RA ➢ High baseline SAA levels may be used for distinguishing non-responders to anti-TNFα therapy
Nissinen et al. (37)	To analyze changes in inflammatory biomarkers in RA patients receiving infliximab	25	RA	ELISA	➢ SAA is a marker of response to infliximab treatment in RA patients
Xu et al. (38)	To explore SAA isoforms in sera from RA patients	169	RA (n=85) HC (n=84)	ELISA	➢ Both SAA1 and total SAA are significantly elevated in RA compared to HC ➢ SAA1/SAA ratio is not significantly different between RA patients and HC
De Seny et al. (39)	To identify RA serum biomarkers by a proteomic approach	103	RA (n=34) AS (n=19) PsA (n=22) OA (n=20) HC (n=16)	SELDI-TOF MS, Western blot	➢ SAA and its truncated forms are significantly elevated in sera from RA patients compared to healthy controls and patients with osteoarthritis
Li et al. (40)	To screen candidate RA associated serum proteins by comparative proteomics	76	RA (n=38) HC (n=38)	2-DE, MS, ELISA	➢ SAA serum concentration is significantly higher in RA patients compared to HC
Seok et al. (41)	To identify biomarkers for improving the accuracy of RA prescreening	80	RA (n=40) HC (n=40)	LC-MS/MS, ELISA	➢ SAA4 is significantly higher in sera with high rheumatoid factor values and may represent a novel prescreening marker for RA diagnosis
Nys et al. (42)	To explore SAA isoforms in different rheumatic diseases	224	RA (n=46: 14 early onset + 20 low activity + 12 high activity) AS (n=30) SLE (n=23) SSc (n=20) OA (n=43) HC (n=62)	LC-MS/MS	➢ SAA2 and SAA1β isoforms are potential RA diagnostic biomarkers prior the onset of symptomatic RA ➢ SAA1α/SAA1β ratio could be used as a marker of disease severity and response to treatment

**Table 2:** Characteristics and results of articles investigating clinical utility of SAA in patients with juvenile idiopathic arthritis.

First author (reference number)	The aim of the study regarding the clinical utility of SAA	Number of patients	Diagnosis (number of patients)	Method used for SAA measuring	Results / Comments
Filipowicz-Sonowska et al. (43)	To investigate SAA levels and its correlations in Polish and American children with JIA and secondary amyloidosis	86	JIA (21 with secondary amyloidosis, 45 without) HC (n=20 with mild infections)	Radioimunno- assay	➢ SAA is significantly elevated in patients with JIA, especially in polyarticular and systemic forms in which amyloidosis occurs more frequently ➢ SAA is not significantly higher in JIA patients with amyloidosis than in those without, therefore is not useful for detecting amyloidosis
Scheinberg et al. (44)	To evaluate clinical utility of measuring SAA levels in JIA	90	JIA (4 with secondary amyloidosis, 96 without)	Radioimunno- assay	➢ SAA levels are significantly correlated with polyarticular and systemic forms as well as with disease activity in patients with JIA, but not with the presence of secondary amyloidosis
Kutulculer et al. (45)	To investigate several biomarkers in assessing disease stage in JIA patients	147	JIA (n=82) FMF (n=35) HC(n=30)	Immunonephelo-metric assay	➢ SAA levels show significant difference between active and remission stages in individuals with JIA or FMF
Cantarini et al. (46)	To investigate SAA as a marker of disease activity in JIA	67	JIA (n=41: 16 polyarticular + 25 oligoarticular) HC(n=26)	ELISA	➢ SAA is a more sensitive marker than ESR and CRP in assessing disease activity in JIA (evaluated as presence and number of active joints- clinically)
Dev et al. (47)	To investigate SAA as a marker of disease activity in JIA	90	JIA (n=50) HC (n=40)	ELISA	➢ SAA is a more sensitive marker than ESR and CRP in assessing disease activity in JIA (evaluated as presence and number of active joints- clinically and USG score)
Miyamae et al. (48)	To explore biomarkers for monitoring and predicting response to therapy in JIA	23	Systemic JIA	SELDI-TOF MS, Immunonephelo-metric assay	➢ SAA is significantly correlated with response to both conventional and biologic therapy ➢ Baseline SAA level may be used for predicting response to therapy

**Table 3:** Characteristics and results of articles investigating clinical utility of SAA in patients with ankylosing spondylitis.

First author (reference number)	The aim of the study regarding the clinical utility of SAA	Number of patients	Diagnosis (number of patients)	Method used for SAA measuring	Results / Comments
Lange et al. (49)	To evaluate SAA as marker of inflammation in AS	72	AS	ELISA	➢ SAA correlates significantly with disease activity in AS patients (assessed by BASDAI score), but is not superior to CRP or ESR
Jung et al. (50)	To evaluate SAA as marker of disease activity in AS patients	76	AS (n=38) HC (n=38)	Immunonephelo-metric assay	➢ SAA is a more sensitive marker than ESR and CRP in assessing disease activity in AS (evaluated by BASDAI score)
De Vries et al. (51)	To evaluate SAA as disease activity marker in AS patients receiving anti-TNF therapy	155	AS	ELISA	➢ SAA correlates significantly with disease activity, response to anti-TNF therapy, and has high predictive value for response to anti-TNF therapy ➢ SAA may be indicator of anti-drug antibodies development
Li et al. (52)	To find a diagnostic marker for AS by proteomic approach	76	AS (n=38) HC (n=38)	2-DE+MALDI-TOF MS, ELISA	➢ SAA may be used as a diagnostic indicator of AS , since it is over-expressed by more than 3-fold in the sera of AS patients compared to healthy controls
Rademacher et al. (53)	To find biomarkers for predicting radiographic progression of AS	117	AS (28 progressors + 89 nonprogressors)	ELISA	➢ SAA levels are significantly elevated in AS patients, but do not correlate with future radiographic progression
Wu et al. (54)	To find serum biomarkers for assessing response to adalimumab in AS patients by proteomic approach	151	AS (n=82) HC (n=69)	Human antibody array, ELISA	➢ SAA1 is significantly correlated with disease activity in AS patients treated with adalimumab ➢ SAA1 significantly correlates with peripheral joint involvement in AS
Liu et al. (55)	To explore serum biomarkers in AS patients by proteomic approach	410	AS (n=192: 164 active + 28 inactive) HC (n=218)	Tandem Mass Tag Proteomics, ELISA	➢ SAA is a sensitive marker for diagnosis and assessing disease activity in AS ➢ SAA1 is even more sensitive marker than total SAA ➢ Combination of CRP and SAA1 increases sensitivity and specificity of CRP alone for diagnosis and assessing disease activity in AS

**Table 4:** Characteristics and results of articles investigating clinical utility of SAA in patients with different types of vasculitis.

First author (reference number)	The aim of the study regarding the clinical utility of SAA	Diagnosis (number of patients)	Method used for SAA measuring	Results / Comments
Ma et al. (56)	To find a potential biomarker for assessing TA activity	TA (n=43: 18 active + 25 inactive), HC (n=20)	ELISA	➢ SAA is significantly higher in patients with active TA than in those with inactive disease, and is significantly higher in both groups compared to HC
Nair et al. (57)	To evaluate SAA as a marker of disease activity and treatment response in TA	TA (n=99: 43 active + 48 inactive + 8 indeterminate), HC (n=40)	ELISA	➢ SAA is a more sensitive biomarker of disease activity and treatment response than CRP or ESR in TA patients
Hocevar et al. (58)	To explore serum biomarkers utility for predicting relapse in GCA patients receiving GCs	GCA (n=68: 31 relapsed + 37 nonrelapsed)	ND	➢ High baseline SAA values are predictors of early relapse in GCA patients treated with GCs ➢ Baseline SAA is better correlated with future relapses than CRP or ESR
Burja et al. (59)	To identify serum biomarkers for monitoring disease activity in patients with GCA	GCA (n=82: 35 relapsed + 47 nonrelapsed), HC (n=46)	Immuno-nephelometric assay	➢ SAA may be used as a marker for GCA diagnosis and disease activity (more sensitive than CRP or ESR)
Dartavel et al. (60)	To evaluate SAA as a marker of disease activity in GCA	GCA (n=80: 21 active + 59 inactive), microbial infections (n=8)	ND	➢ SAA is significantly different between GCA patients with active and inactive disease ➢ SAA levels are not different between patients with active GCA and microbial infections
Van Sleen et al. (61)	To find biomarkers for predicting disease course in GCA and for monitoring response too therapy	GCA (n=41) HC (n=33) microbial infections (n=13)	ELISA	➢ SAA is significantly elevated in GCA compared to HCs, but not compared to patients with infection ➢ SAA correlates with disease activity in GCA patients receiving GCs ➢ Baseline SAA levels do not have a predictive value for disease course in GCA
Mitani et al. (62)	To find potential biomarkers of coronary artery lesions late after KD	KD (n=65: 20 with coronary artery lesions, 45 without)	Immuno-nephelometric assay	➢ SAA is significantly higher in patients with coronary artery lesion persistence late after KD than in patients with regressed or without lesions
Whitin et al. (63)	To find a serum biomarker for diagnosis of KD	KD (n=68) Febrile controls (n=61)	SELDI-TOF MS + hybrid MS immunoassay	➢ A truncated form of SAA (7860 Da) may be a diagnostic marker for KD
Purevdorj et al. (64)	To find a serum biomarker for diagnosis of HSP	HSP (n=127), Infections (n=110), HC (n=121)	ELISA	➢ SAA is a sensitive and specific diagnostic biomarker for HSP (better than CRP)
Kuret et al. (65)	To evaluate serum biomarkers for diagnosis of IgA vasculitis	IgA vasculitis (n=62) HC (n=53)	Immuno-nephelometric assay	➢ SAA is significantly increased in adult IgA vasculitis and may be used as a diagnostic marker

**Table 5:** Characteristics and results of articles investigating clinical utility of SAA in patients with sarcoidosis.

First author (reference number)	The aim of the study regarding the clinical utility of SAA	Diagnosis (number of patients)	Method used for SAA measuring	Results /Comments
Rothkrantz-Kos et al. (66)	To evaluate the clinical usefulness of SAA for assessing sarcoidosis severity	Sarcoidosis (n=144: 73 untreated + 71 treated) HC (n=282)	Immunonephelo-metric assay	➢ SAA does not correlate with the disease severity in sarcoidosis
Miyoshi et al. (67)	To identify marker predictive of increased parenchymal infiltration in sarcoidosis	Sarcoidosis (n=43)	Immunoturbidi-metric assay	➢ SAA concentration at diagnosis is not predictive of increased parenchymal infiltration later in sarcoidosis
Salazar et al. (68)	To evaluate SAA as a marker of disease activity in patients with sarcoidosis	Sarcoidosis (n=85: 40 active + 45 inactive)	ELISA	➢ SAA is significantly higher in sarcoidosis patients with active disease than in those with inactive disease
Bargagli et al. (69)	To investigate possible benefits of SAA as a biomarker in sarcoidosis	sarcoidosis (n=55) HC (n=24)	2-DE+western blot, ELISA	➢ SAA and SAA1 may be used as a diagnostic marker for sarcoidosis, marker of disease activity and a predictor of severe disease (requiring steroid therapy)
Gungor et al. (70)	To find a sensitive marker of sarcoidosis disease activity	Sarcoidosis (n=48: 37 active + 11 inactive) HC (n=20)	ELISA	➢ SAA can be used as a marker of sarcoidosis activity since it correlates with disease activity (better than CRP)
Zhang et al. (71)	To find a serum marker for differential diagnosis of sarcoidosis from other lung diseases	Sarcoidosis (n=37) Tuberculosis (N=20) Other pulmonary diseases(n=20) HC (n=20)	MALDI-TOF MS, ELISA	➢ SAA levels are significantly higher in sarcoidosis patients compared to other lung diseases and can be used as a diagnostic marker for sarcoidosis ➢ Truncated SAA forms might be an even more specific diagnostic marker for sarcoidosis
Enyedi et al. (72)	To evaluate markers for differential diagnosis of sarcoidosis from other lung diseases	Sarcoidosis (n=69) Other lung diseases (n=35)	Immunonephelo-metric assay	➢ SAA does not significantly differ between patients with biopsy-proven sarcoidosis and biopsy negative patients

**Table 6:** Characteristics and results of articles investigating clinical utility of SAA in patients with systemic sclerosis, systemic lupus erythematosus or psoriatic arthritis.

First author (reference number)	The aim of the study regarding the clinical utility of SAA	Number of patients	Diagnosis (number of patients)	Method used for SAA measuring	Results / Comments
Brandwein et al. (73)	To explore SAA as a marker of SSc severity	74	SSc (n=62) HC (n=12)	Radioimmuno-assay	➢ SAA is significantly correlated with SSc severity (assessed by extension of skin thickening)
Lakota et al. (74)	To determine clinical correlations of SAA in patients with SSc	227	SSc (n=129) HC (n=98)	ELISA	➢ SAA significantly correlates with pulmonary function and is a sensitive marker of pulmonary involvement in SSc
Lis Swiety et al. (75)	To evaluate SAA in SSc patients in relation to skin and pulmonary involvement	48	SSc (n=33: 18 early dSSc + 15 late dSSc) HC (n=15)	ELISA	➢ SAA is superior to CRP as a biomarker of pulmonary involvement in SSc
Wang et al. (76)	To investigate correlation between SAA levels and disease activity in SLE	284	SLE (n=135: 52 active + 83 inactive disease) HC (n=149)	Immunonephelometric assay	➢ SAA is independently significantly correlated with SLE disease activity (assessed by SLEDAI score)
Boyd et al. (77)	To evaluate correlations between serum biomarkers and disease activity in psoriatic arthritis	45	PsA (n=45)	ELISA	➢ SAA is the marker with the highest correlation to the disease activity in PsA patients (compared to 15 measured biomarkers including CRP)

**Table 7:** Characteristics and results of articles investigating clinical utility of SAA in patients with systemic autoinflammatory diseases.

First author (reference number)	The aim of the study regarding the clinical utility of SAA	Diagnosis (number of patients)	Method used for SAA measuring	Results / Comments
Duzova et al. (78)	To evaluate SAA in detecting subclinical activity in FMF and for guiding colchicine therapy	FMF (n=183, attack-free period)	ND	➢ SAA level remains high even between attacks in children with FMF, therefore can be used for detecting subclinical inflammation ➢ SAA monitoring should be used as a guidance for therapy adjustment
Lachmann et al. (79)	To evaluate inflammatory activity in FMF patients	FMF (n=43) MEFV mutation carriers (n=67) HC (n=50)	Immunonephelometric assay	➢ SAA is significantly elevated during FMF attacks and remains increased between attacks ➢ SAA is higher among asymptomatic heterozygous carriers of MEFV mutations compared to HC, potentially leading to development of amyloidosis (phenotype II) ➢ SAA level monitoring should be used for assessment of and reinforcing patients compliance for therapy
Berkun et al. (80)	To evaluate SAA as a marker for diagnosis and therapy adjustment in FMF	FMF (n=204)	Immunonephelometric assay	➢ SAA level is an indicator of subclinical inflammation in FMF patients, a guidance for colchicine adjustment and a marker for differentiating noncompliance and nonresponse to colchicine
Yalcinkaya et al. (81)	To evaluate the clinical utility of SAA in FMF patients with amyloidosis	FMF (n=51: 36 without amyloidosis + 15 with amyloidosis) MEFV mutation carriers (n=39) Other chronic inflammatory diseases (n=39) Infections (n=20) HC (n=19)	ELISA	➢ SAA is elevated during acute attacks and remains above reference range even during attack-free periods in FMF patients ➢ SAA is not significantly higher in FMF patients with a higher potential for developing amyloidosis than in those without, so cannot be used for predicting amyloidosis
Kallinich et al. (82)	To find biomarkers of inflammation in FMF	FMF (n=52: 28 without attack, 19 during attack, 5 mutation carriers)	Immunonephelometric assay	➢ SAA concentration is significantly elevated in FMF patients at diagnosis, during attacks, in between the attacks and in MEFV mutation carriers
Lofty et al. (83)	To investigate the clinical utility of monitoring SAA levels in FMF	FMF (n=71)	ELISA	➢ SAA is elevated even during attack-free periods in FMF and, therefore, should be used for detecting subclinical inflammation
Cakan et al. (84)	To determine the capability of SAA in differentiating attacks of FMF from acute febrile infections	FMF (n=28) Acute respiratory infection (n=28)	Immunonephelometric assay	➢ SAA is significantly higher in acute FMF attack than in acute febrile respiratory infection
Bilginer et al. (85)	To find biomarkers for predicting atherosclerosis in FMF patients	FMF (n=70, attack-free period) HC (n=50)	ELISA	➢ SAA is significantly higher in FMF patients even in attack-free periods compared to HC ➢ SAA is significantly correlated with CIMT (an early predictor of atherosclerosis)
Mohamed et al. (86)	To explore relationship between SAA and CIMT in FMF patients	FMF (n=45, attack-free period) HC (n=40)	ELISA	➢ SAA is significantly elevated in FMF patients compared to HC ➢ SAA is significantly correlated with attack severity and CIMT in FMF patients
Sargsyan et al. (87)	To find biomarkers of vascular involvement in FMF patients	FMF (n=102: 50 with vascular disease, 52 without)	ELISA	➢ SAA is significantly higher in FMF patients with vascular involvement than in those without
Aygunduz et al. (88)	To evaluate SAA as a marker of disease activity in BD	BD (n=43: active 20 + inactive 23) HC (n=27)	ELISA	➢ SAA is significantly elevated in patients with BD and correlates with disease activity
Cantarini et al. (89)	To evaluate potential correlations between circulating biomarkers and clinical activity of BD	BD (n=27) HC (n=35)	ELISA	➢ SAA is not significantly correlated with disease activity in BD ➢ SAA is an indicator of skin involvement in BD
Vitale et al. (90)	To evaluate SAA as a marker of disease activity in BD	BD (n=26)	ELISA	➢ SAA serum levels higher than 30, 50 and 150 mg/L are significantly associated with the occurence of oral aphthosis, neurological and ocular involvement, respectively
Sota et al. (91)	To explore potential values of SAA as a biomarker in patients with BD	BD (n=64)	ELISA	➢ SAA levels do not correlate with disease activity in BD ➢ SAA levels >200 mg/L are significantly associated with major organ involvement ➢ SAA levels >150mg/L are associated with ocular, skin and mucosal involvement in BD
Lee et al. (92)	To evaluate SAA as a biomarker in intestinal BD by proteomic analysis	Intestinal BD (n=64: 9 mild + 35 moderate + 20 severe) HC (n=56)	2-DE + MALDI-TOF/TOF MS, ELISA	➢ SAA is significantly elevated, but is not correlated with disease severity in patients with intestinal BD
Hawkins et al. (93)	To evaluate SAA in monitoring response to anakinra (anti-IL-1R antibody) in MWS patients	MWS (n=3)	Immunonephelometric assay	➢ SAA is significantly reduced after initiation of anakinra treatment in MWS as well as the clinical symptoms, therefore can be used for monitoring response to biologic therapy
Scarpioni et al. (94)	To evaluate SAA in monitoring response to canakinumab in MWS patients	MWS (n=2)	Immunonephelometric assay	➢ SAA is significantly reduced after initiation of canakinumab treatment in MWS as well as the clinical symptoms, therefore can be used for monitoring response to therapy
Hoffman et al. (95)	To evaluate SAA in monitoring response to rilonacept in patients with CAPS	FCAS (n=95) MWS (n=3) FCAS/MWS (n=3)	ND	➢ SAA levels are significantly reduced after initiation of rilonacept therapy and remain low during long-term follow-up
Goldbach-Mansky et al. (96)	To evaluate SAA in monitoring response to rilonacept (anti-IL-1R therapy) in patients with CAPS	FCAS (n=5)	ND	➢ SAA significantly correlates with disease activity and response to anti-IL-1 therapy in FCAS patients
Wiken et al. (97)	To evaluate SAA in monitoring response to anakinra in patients with CAPS	NOMID (98) MWS (7)	Immunonephelometric assay	➢ SAA is significantly reduced after initiation of anakinra treatment in CAPS as well as the clinical symptoms and the development of anti-drug antibodies does not effect either

**Table 8:** Characteristics and results of articles investigating clinical utility of SAA in patients with AA amyloidosis.

First author (reference number)	The aim of the study regarding the clinical utility of SAA	Diagnosis (number of patients)	Method used for SAA measuring	Results / Comments
Falck et al. (6)	To investigate the role of monitoring SAA levels in RA patients with secondary amyloidosis	RA with AA amyloidosis (n=20)	Radioimmuno-assay	➢ Mean SAA levels are significantly correlated with the change in renal function (creatinine clearance), therefore can be used for predicting renal deterioriation in amyloidosis
Ishii et al. (99)	To investigate SAA serum levels and SAA genotype in RA patients with amyloidosis	RA (n=217: 200 without + 17 with amyloidosis)	ND	➢ SAA levels are significantly higher in RA patients with amyloidosis than in those without ➢ SAA1.3 allel is a risk factor for developing amyloidosis in Japanese population
Migita et al. (100)	To investigate correlation between SAA concentrations and the presence of amyloidosis in RA patients	RA (n=56: 18 with amyloidosis + 38 without)	Immuno-nephelometric assay	➢ SAA concentration is not correlated with the presence of amylodiosis in RA patients ➢ The ratio of SAA-derived fragments to total SAA is significantly higher in patients with amyloidosis
Gorlier et al. (101)	To find a diagnostic marker for AA amyloidosis in FMF patients	FMF (n=56: 50 without + 6 with amyloidosis)	ND	➢ Mean SAA is not significantly correlated with the presence of AA amyloidosis, therefore is not clinically useful for detecting amyloidosis in FMF
Lachmann et al. (102)	To evaluate potential benefits from monitorig SAA levels in AA amyloidosis	AA amyloidosis (n=374, different underlying diseases)	Immuno-nephelometric assay	➢ Median SAA level is an indicator of changes in renal function, prognostic factor and indicator of death risk in AA amyloidosis ➢ SAA monitoring should be used for therapy guidance in patients with AA amyloidosis
Gilmore et al. (103)	To assess amyloid load in relation to SAA levels in amyloidosis	AA amyloidosis (n=80, different underlying diseases)	ELISA	➢ Median SAA level is significantly correlated with changes in amyloid load and long-term survival in patients with secondary amyloidosis
Perry et al. (104)	To investigate the effect of etanercept on SAA levels in patients with amyloidosis	AA amyloidosis (n=9, different underlying diseases)	ND	➢ SAA serum levels may be used for monitoring response to etanercept in patients with AA amyloidosis
Nakamura et al. (105)	To investigate etanercept effects on SAA levels and disease activity in amyloidosis	RA with amyloidosis (n=14)	ND	➢ Etanercept induced SAA decrease is followed by decrease in disease activity in amyloidosis patients, therefore SAA may be used for monitoring response to etanercept
Mijagawa et al. (106)	To assess effects of tocilizumab on SAA levels in AA amyloidosis	RA with amyloidosis(n=5)	ND	➢ Tocilizumab induced SAA decrease is associated with clinical improvement in amyloidosis, therefore SAA may be used for monitoring response to tocilizumab
Lane et al. (107)	To study efficacy of tocilizumab in AA amyloidosis	AA amyloidosis (n=14, different underlying diseases)	Immunonephelo-metric assay	➢ Decrease in SAA levels in amyloidosis patients receiving tocilizumab is associated with decreased proteinuria and amyloid regression, therefore SAA can be used as a marker of response to tocilizumab
Okuda et al. (108)	To compare the effects of anti-IL-6 and anti-TNFα therapy on SAA levels in AA amyloidosis	AA amyloidosis (n=42, different underlying diseases)	ND	➢ Decrease in SAA levels and disease activity is significantly greater in patients receiving anti-IL-6 than in those receiving anti-TNFα therapy

**Table 9:** Characteristics and results of articles investigating clinical utility of SAA in patients with COVID-19.

First author (reference number)	The aim of the study regarding the clinical utility of SAA	Number of patients (n)	Results / Comments
Xu et al. (109)	To explore changes in serum biomarkers in COVID-19 patients	187	➢ SAA levels are significantly increased in COVID-19 patients and may be used as a diagnostic marker ➢ The mean SAA concentration in critically-ill patients is significantly higher than in mild-ill patients, therefore may be used as a marker of disease severity in COVID-19
Shi et al. (110)	To find serum biomarkers of disease severity in COVID-19	114	➢ SAA may be used as a marker of disease severity in COVID-19 patients
Wang et al. (111)	To evaluate biomarkers in assessing COVID-19 severity	143	➢ SAA is significantly associated with COVID-19 severity ➢ SAA levels above 100 mg/L are indicative of disease progress to the critical stage
Li H et al. (112)	To evaluate SAA as a marker of COVID-19 severity and prognosis	132	➢ SAA is a sensitive marker of COVID-19 severity ➢ Dynamic changes in SAA level are significantly correlated with clinical outcome of COVID-19
Li X et al. (113)	To reveal a predictor of fatal outcome in COVID-19	25	➢ SAA might be a predictor of fatal outcome in patients with COVID-19
Mo et al. (114)	To find a serum biomarker with a predictive value for COVID-19 prognosis	118	➢ SAA levels correlate with COVID-19 severity ➢ SAA is an independent predictor of severe COVID-19 with accuracy of 89.1% at the cut-off value of 122.9 mg/L

## SAA-Related Genes and Proteins

The SAA gene family is located on the short arm of chromosome 11 (11p15.1). It contains four genes, namely *SAA1*, *SAA2*, *SAA3* and *SAA4* (115). All the genes consist of 4 exons and 3 introns, and their initial transcripts have 18 aa signal sequence that is removed in the serum proteins. Within the SAA gene cluster, only the *SAA1* and *SAA2* genes encode an acute phase serum proteins (SAA1 and SAA2 isotypes) with approximately 95% sequence identity, which are coordinately induced in response to inflammation (116). *SAA3* contains an early stop codon suggesting it is a non-translated pseudogene (117). The corresponding protein of the *SAA4* gene is constitutively synthesized, meaning it is not induced in the acute phase response (118). The inducible SAA isoforms, SAA1 and SAA2, are termed acute-phase SAA.

SAA has several allelic variants (α, β, γ in *SAA1* and α, β in *SAA2*). Two single nucleotide polymorphisms (SNPs) within the exon 3 of the *SAA1* gene generate three common isoformes: SAA1α (52Valine/57 Alanine), SAA1β (52Alanine/57 Alanine) and SAA1γ (52Alanine/57 Valine). Some of these variants contribute to the susceptibility to AA amyloidosis. In particular, *SAA1α* allele is a risk factor for developing AA amyloidosis in Caucasian (119–121). Contrarily, in the Japanese population this allelic variant has protective properties, while *SAA1γ* allele carries a higher risk of developing AA amyloidosis (122–124). Blank et al. (119) reported a 100% incidence of *SAA1α/α* genotype among patients with idiopathic AA amyloidsosis. Moreover, another SNP in the *SAA1* gene at position -13 in the 5’ regulatory region (promoter region), is associated with the AA amyloidosis occurrence in both Japanese and Caucasian rheumatoid arthritisnbsp;patients, which might explain the discrepancy between previous reports (125, 126). The latter SNP also affects the SAA transcription with the -13T allele having greater activity (127).

SAA transcription can be up-regulated by several cytokines including the tumor necrosis factor alpha (TNFα), IL-1β and IL-6 (128). TNFα and IL-1β activate the nuclear factor-kappa B (NF-κB) site. IL-6 binds to a transmembrane G-coupled protein 130 (gp130) leading to the activation of Janus kinase 2 (JAK-2), which results in the recruitment of the signal transducer and activator of transcription 3 (STAT3), finally resulting in an impressive SAA gene transcription (129, 130). However, a weak expression of SAA messenger RNA (mRNA) is induced by the stimulation with IL-6 alone, whereas almost no expression is induced by the stimulation with TNFα or IL-1β alone. On the other hand, the synergistic induction of SAA mRNA has been observed by a co-stimulation with IL-6 and TNFα or IL-1β (131). It seems that the activation of STAT3 by an IL-6 stimulated JAK is essential for the production of SAA and the supplementation of NF-κB activity stimulated by TNFα or IL-1β strengthens the SAA expression (132). This evidence is important for the therapeutic effects of monoclonal antibodies: JAK inhibitors (tofacitinib) and anti-IL-6 receptor antibodies (tocilizumab and sarilumab) almost completely inhibit the expression of the SAA mRNA, whereas the IL-1 antagonists (anakinra) and TNFα antibodies (infliximab, adalimumab, etanercept) achieve only a partial inhibition (108, 130). However, SAA mRNA translation is 10-fold lower than the rate of mRNA synthesis due to post-transcriptional regulation. SAA mRNA undergoes poly(A) tail shortening over time, a posttranscriptional event that has been functionally coupled to gene expression and translation. These post-transcriptional mechanisms are only partially explained, suggesting possible epigenetic modifications (133, 134). The half-life time of SAA (~35h) is significantly shorter than that of CRP (~47h) (4). Interestingly, half-life time of their mRNAs (SAA mRNA~8.5h, CRP mRNA~2.5h) indicate that SAA mRNA stability is substantially greater than the CRP mRNA. Taken all together, it seems that CRP expression is regulated mainly at the transcriptional level, while post-transcriptional mechanisms are involved in the regulation of SAA (133, 135). This is somewhat in line with the notion that SAAcan be readily produced locally by synovial cells in joints of RA patients (20, 136). Moreover, Thorn et al. (128, 137) detected a putative glucocorticoid response element (GRE) functionally active in the *SAA1* gene, whereas it was disrupted in the *SAA2* gene. A paradox of an anti-inflammatory drug inducing the pro-inflammatory mediator was confirmed by De Seny et al. (20) who demonstrated glucocorticoid-induced SAA secretion in human primary joint cells.

## SAA in Rheumatic Diseases

The role of SAA in pathogenesis of rheumatic diseases has been most extensively investigated in RA, a IRD prototype, characterized by synovial inflammation leading to a cartilage destruction. The finding that the SAA concentration might be even higher in synovial fluid than plasma, led to discovery of local SAA production by rheumatoid synovial cells (20, 29, 138). Connolly et al. demonstrated a SAA-induced leukocyte migration and tissue infiltration, angiogenesis and inflammation in synovial cells in rheumatoid arthritis (98, 139). Moreover, SAA exhibits cytokine-like properties and can induce synthesis and secretion of several proinflammatory cytokines, including TNFα, IL-6 and IL-1ß (140, 141). SAA plays a pathogenic role in joint leading to the cartilage destruction by activating multiple receptors, including N-formyl peptide receptor-like 1 (FPRL1, also called lipoxin A4 receptor) (142, 143), scavenger receptor class B type 1 (SR-B1) (144, 145), Toll-like receptor 4 (TLR4) (146, 147), Toll-like receptor 2 (TLR2) (148–151) and receptors of advanced glycation end products (RAGE) (147, 152). The expression of these receptors is increased in RA synovial tissue and mediates SAA-induced proinflammatory and angiogenic effects by the activation of MAPKs and NF-κB (153). Moreover, SAA stimulates the production of matrix metalloproteinases (MMPs) by chondrocytes and synovial fibroblasts (154–156). Stimulation of these cartilage-degrading proteinases contributes to the chronic tissue injury in arthritis. Matrix metalloproteinase-3 (MMP-3) is found highly concentrated in the synovial fluid as well as in the serum of RA patients and correlates with progression of erosion in RA (21, 157). MMP-3 production is simultaneously up-regulated by the proinflammatory cytokines IL-1β, TNFα and IL-17. Cytokine- and SAA-driven production of MMP-3 in the rheumatoid joint appears to be a key mediator of the cartilage destruction. Furthermore, SAA induces pentraxin 3 (PTX3) in rheumatoid synoviocytes. PTX3 is also an acute-phase reactant involved in amplification of the inflammatory response. This loop seems to involve N-formyl peptide receptor ligand-1 (FRLP-1) (158).

**Figure 2:**
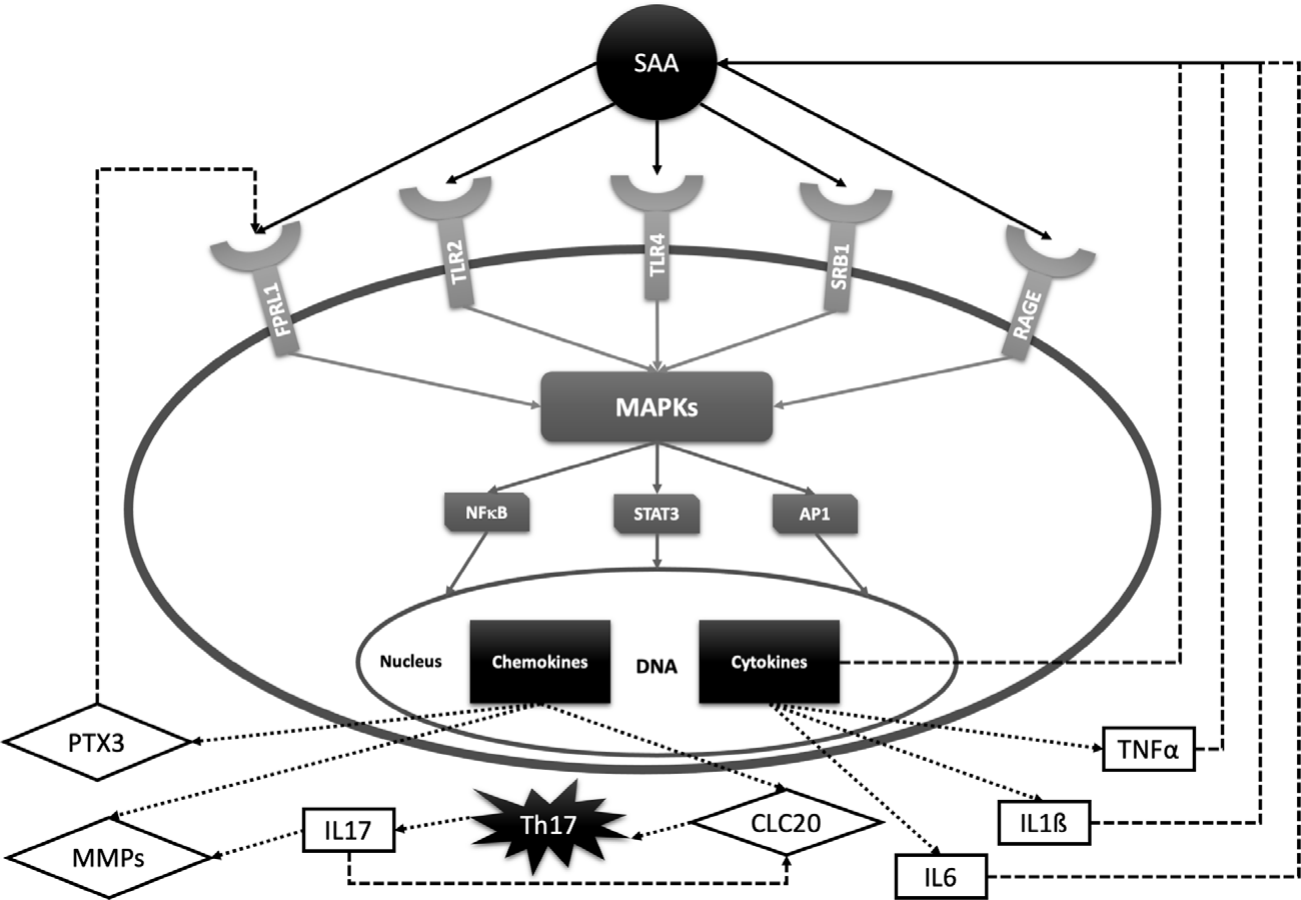
SAA signal transduction pathways and feedback loops with relevance for rheumatic diseases. SAA activates several cell receptors including Toll-like receptors 2 and 4 (TLR2, TLR4), formyl peptide receptor -like 1 (FPRL1), scavenger receptor class B type 1 (SRB1) and receptors of advanced glycation end products (RAGE). SAA receptors share common properties in activating protein kinases (MAPKs) and transcription factors such as nuclear factor kappa B (NF-kB), signal transducer and activator of transcription 3 (STAT-3) and activator protein 1 (AP-1). These factors promote transcription of interleukin 6 (IL-6), interleukin 1ß (IL-1ß), tumor necrosis factor α (TNFα), matrix metalloproteinases (MMPs), chemokine ligand 20 (CLC20), pentraxin 3 (PTX3), etc. IL-6, IL-1ß and TNFα stimulate SAA production, while PTX3 promotes inflammation by activating FPRL-1. CLC20 recruits Th17 cells which stimulate transcription of cartilage-degrading MMPs and CLC20 chemokine by producing IL-17.

A new subset of interleukin 17 (IL-17) producing T helper cells (Th17 cells) has been recently reported to play a critical role in inflammatory joint diseases including RA, AS and psoriatic arthritis (PsA) (159–165). In contrast to the other effector T-cell subsets, Th17 cells express the IL-23 receptor (IL-23R) on their membrane and are dependent on IL-23 for their survival, expansion and cytokine production (159). In addition, Th17 cells express the chemokine receptor 6 (CCR6) on their membrane which can be activated by the chemokine ligand 20 (CLC20) (160). CLC20 acts as a chemo-attractant on Th17 cells and stimulates IL-17 production. SAA is a potent inducer of both IL-23 and CCL20 in synovium and, consequently, induces Th17 polarization from CD4 + T cells and IL-17 production (161, 162). Furthermore, IL-17 also up-regulates the expression of CCL20 (166). Taken together, SAA sustains the chronic inflammation by contributing to the recruitment of Th17 cells to the inflamed synovium. Although serum and synovial fluid IL-17 levels in RA patients are significantly elevated (163, 164), results of clinical trials with anti-IL-17 antibodies have been discouraging. On the other hand, IL17 blockade is highly effective in AS and PsA (166, 167). This may be due to a non-IL-23 dependent IL-17 production in innate immune cells, which can contribute to the pathogenesis of these diseases (166). **Figure 2** summarizes the described SAA signal transduction and feedback pathways.

Recent studies (168–170) have shown that SAA also induces the synthesis of pro-IL-1β and activation of the NOD-like receptor family, pyrin domain containing 3 (NLRP3) inflammasome and subsequent activation of caspase-1 which converts pro-IL-1β to its active form IL-1β, suggesting a further link between SAA and systemic autoinflammatory disease. Activation of the inflammasome cascade has one of the key roles in initiation of the whole immune system. This recently discovered connection, in addition to the above mentioned SAA immunomodulatory pathways, emphasizes the importance of SAA in the pathogenesis of rheumatic diseases. Proteins that are mutated in autoinflammatory diseases mediate the regulation of NF-κB activation, cell apoptosis, and IL-1β secretion through cross-regulated signaling pathways. Since almost all clinical manifestations associated with inflammasome dysregulation are due to an inappropriate and/or excessive release of IL-1β, targeted IL-1β blockade is the mainstay of treatment, and its remarkable efficacy is well established. Biological therapies such as anakinra (recombinant IL-1 receptor antagonist) and canakinumab (monoclonal anti-IL-1β antibody) are both licensed for several autoinflammatory diseases.

In 1974, Husby et al. identified amyloid protein A as a proteolytic derivate of SAA by a sequence analysis of the protein purified from amyloid deposits. Amyloid protein A is a 76 amino acids N-terminal derivate of SAA (171). The aberrant processing of SAA in macrophage lysosomes leads to accumulation of newly formed AA amyloid fibrils and development of AA amyloidosis (172, 173). The generation of SAA N-terminal fragments has been related to the activity of MMPs (174). MMP1 has a putative cleavage site next to the site of SAA1 amino acid substitutions at positions 52 and 57 (175). SAA1α has a higher affinity for cleavage by MMP1 than SAA1β or SAA1γ consequently leading to a larger amounts of amyloidogenic truncated SAA forms (175). These data indicate that susceptibility to N-terminal cleavage at residue 57 might be related to a higher risk of developing amyloidosis.

In blood circulation SAA associates with high density lipoproteins (HDL), where it replaces apolipoprotein A1. During the acute phase response, SAA constitutes up to 87% of the apolipoprotein content of HDL particles. Incorporation of SAA into the HDL particle leads to a structural modification with a consequent functional deficiency. These so-called proinflammatory HDLs (piHDLs) are characterized by reduced capacity for reverse cholesterol transport, increased oxidation of low density lipoproteins (LDLs) and reduced inhibition of monocyte chemoattractant protein 1 (MCP-1) production in vascular smooth muscle cells. These changes result in an increased atherogenic potential (176, 177). Recent studies (178, 179) have proposed piHDLs as biomarkers of disease activity in IRD. Additionally, high expression of SAA mRNA by several cell types in atherosclerotic lesions suggests a role in the pathogenesis of atherosclerotic plaques (180). The earliest phase of atherogenesis involves vascular endothelial cell (EC) dysfunction. The SAA-induced pro-inflammatory cytokine TNFα stimulates the expression of adhesion molecules on ECs and stimulates the production of tissue factor (TF) which promotes the formation of atherosclerotic plaque (181, 182). SAA-treated ECs show a significantly increased expression of TNFα, TF and vascular endothelial growth factor (VEGF) (183). Pharmacological blockade of SAA receptors, including FPRL1, TLR2/4 and RAGE, inhibits SAA-mediated pro-atherogenic effects in ECs. However, the pharmacological inhibition is only partial in contrast to adding isolated human HDL which almost completely abrogates SAA-induced pro-atherogenic activity (183, 184). HDL reduces SAA’s bioavailability, but other mechanisms for modulating SAA pro-atherogenic activities in EC are also possible. For instance, circulating HDL may indirectly inactivate membrane SAA receptors by membrane cholesterol level modulation (185). Therefore, SAA:HDL blood ratio may be of a critical importance for HDL’s ability to protect ECs from SAA pro-atherogenic activities. Furthermore, SAA up-regulates the expression of TLR2 in EC, suggesting a positive feedback loop (186). On top of all that, SAA significantly decreases endothelial nitric oxide (NO) synthase levels, NO bioavailability and the activity of internal antioxidant enzymes catalase and superoxide dismutase in ECs, leading to an increased superoxide radical anion production, impaired NO activity and, consequently, endothelial dysfunction and atherosclerotic plaque formation (187). Intriguingly, atherosclerotic cardiovascular diseases are the primary cause of premature death in patients with rheumatic diseases.

## SAA in Rheumatoid Arthritis

SAA can be used as a diagnostic marker for RA since its serum levels are significantly elevated in RA patients compared to healthy controls and patients with osteoarthritis (OA) (5, 9–11). SAA levels are increased in RA compared to OA patients not only in the serum, but also in synovial fluid, as a result of a local production (12, 20, 29). A number of researches demonstrated a significant correlation between SAA serum concentration and RA disease activity (9, 10, 13). SAA is a more sensitive marker of disease activity in RA than CRP or ESR (11, 14–16, 21), even during pregnancy (17). Chambers et al. were the first to report an increased SAA level in RA patients with normal CRP levels in 40% of the patients included in their study (14). Authors considered sex, age, disease duration as well as therapy differences between groups in consideration, but did not find a significant divergence that could influence results. Subsequent studies confirmed the usefulness of SAA for detecting subclinical inflammation, even in patients with CRP within the reference values (18, 19, 22, 29). Hwang et al. (18) and Connolly et al. (23) proposed SAA for monitoring RA disease activity in patients receiving anti-TNFα therapy, since anti-TNFα therapy reduced CRP even without reduction in disease activity, while SAA was less affected. This may be a result of synthesis under the influence of different cytokine combinations.

Furthermore, considerably increased SAA levels (>520 mg/L) in patients with recent onset arthritis can distinguish patients with a final diagnosis of RA from those with persistent undifferentiated arthritis (15). In addition, baseline SAA levels, contrarily to CRP or ESR, are independently correlated with RA radiographic progression at 1-year (23). Wild et al. (19) performed a multivariate analysis of 32 biomarkers (including SAA and CRP), out of 131 initially considered, which were subjected to three inclusion criteria: discrimination between RA patients and healthy controls, ability to identify anti-cyclic citrullinated peptide antibody (anti-CCP) negative RA patients and specificity for RA. Among the tested biomarkers, only the combination of SAA and anti-CCP increased the sensitivity of anti-CCP alone (80.1% vs. 75.8%, respectively), although this was followed by a drop in specificity (94% for anti-CCP alone vs. 86.6% for SAA+ anti-CCP). Moreover, SAA is incorporated in the widely used multi-biomarker disease activity (MBDA) test developed by Centola et al. (24) for RA activity assessment and discrimination of patients with a low disease activity from those with a moderate or high disease activity. The group tested 130 biomarkers for estimating RA activity in samples obtained from 702 patients and finally included only 12 biomarkers in MBDA score. While most of the MBDA score biomarkers can predict either Swollen Joint Count (SJC28), Tender Joint Count (TJC28) or Patient Global Assessment (PGA), SAA predicts all of them. Recently, Ma et al. (25) investigated the utility of the MBDA score and its individual components for predicting outcomes in patients who are in a stable low disease activity. They found that the baseline SAA level can be used for predicting remission over 12 months. The average of 3 measurements obtained over the first 6 months had an even better predictive value. Moreover, baseline SAA concentrations were significantly higher in patients with low disease activity than patients in total remission. Therefore, SAA may be used for detecting subclinical inflammation and for predicting remission at 1 year in patients with RA.

SAA has also been correlated with cardiovascular disease in RA patients. Wong et al. (26) found that the decreased arterial elasticity in RA patients, which may precede atherosclerosis, is significantly inversely correlated with SAA concentration. Another study reported a trend of association between SAA levels and severity of coronary atherosclerosis in patients with RA (27). Along with a significant correlation between SAA and cardiovascular involvement, Targonska-Stepniak et al. found a significant correlation with renal involvement (assessed by serum levels of cystatin C—an early marker of chronic kidney disease) (13).

Numerous researches verified SAA as an indicator of response to therapy in RA patients. Kullich et al. investigated the effects of leflunomide, a conventional disease-modifying antirheumatic drug (DMARD) therapy in RA (28). Their *in vivo* results with significant reduction of SAA and MMP levels after 6 months of leflunomide therapy confirmed a previously reported suppressive effects of leflunomide *in vitro* (188). Targonska-Stepniak et al. (22) also investigated the influence of leflunomide and found that, in spite of a reduction in disease activity and other laboratory inflammatory markers including CRP and ESR, the mean SAA concentration at 12 months of leflunomide treatment did not significantly differ from the SAA level at the start of treatment, revealing an ongoing subclinical inflammation. More recently, Boeters et al. (30) revealed a significant association between higher SAA levels (>3 ug/ml) at disease onset and achievement of sustained-DMARD-free remission (defined as the absence of synovitis that sustained after discontinuation of all DMARD therapy to at least 1 year) suggesting a predictive value of SAA. Migita et al. (130) demonstrated that Janus kinase inhibition down-regulates IL-6 induced SAA expression in rheumatoid synovium. In the subsequent study (31) they validated SAA as a sensitive biomarker of response to tofacitinib (Janus kinase inhibitor) in patients with active RA. Tofacitinib reduced both IL-6 and SAA serum levels. In patients who experienced a post-treatment SAA normalization, the decrease of disease activity was greater compared to those with persistently high levels. This finding suggests that SAA may be used for monitoring response to tofacitinib in RA patients, although the caution is needed when interpreting the results, since only 14 patients were included in the study, and only 4 received tofacitinib as monotherapy. Finally, many researchers investigated response to anti-TNFα antibodies in RA patients by monitoring SAA levels. Visvanathan et al. (32) reported that the reduction in serum level of SAA at week 4 after initiation of golimumab therapy in RA patients correlates significantly with clinical improvement at week 16, suggesting the use of SAA for predicting the clinical response. Doyle et al. (33) validated SAA as a potential biomarker for evaluating response to golimumab in RA patients but did not found clinically relevant correlation between baseline SAA levels and clinical improvement at week 24 of golimumab therapy. Furthermore, the clinical utility of SAA for assessing response to adalimumab in RA patients has been validated by Kobayashi et al., Berner Hammer et al. and Gabay et al. (34–36), while Nissinen et al. (37) reported SAA levels to be significantly correlated with clinical improvement in RA patients treated with infliximab. In addition, Gabay et al. (36) compared effects of adalimumab and sarilumabon serum biomarkers in a large cohort (n=307) of RA patients. They found a significant greater decrease of SAA at week 24 in responders than non-responders in adalimumab group. Although total SAA reduction was greater in sarilumab group, the association between clinical efficacy and SAA reduction was not found in this group, suggesting that a direct effect of IL-6 blockade on SAA production is independent of its effect on disease activity. In addition, this research reported a significant predictive value of high baseline SAA levels for a better clinical response to sarilumab than adalimumab. Further studies with a longer follow-up are needed for validating and expanding these results.

Lately, quantitative methods for measuring different SAA isoforms and proteomic techniques for exploring clinical relevance of these isoforms are being developed. Xu et al. (38) developed ELISA for SAA1 and investigated levels and ratios of SAA1 in total SAA in healthy subjects and RA patients. They found that both SAA1 and total SAA are significantly elevated in RA patients compared to healthy subjects, although the SAA1/SAA ratio did not differ between the two groups. De Seny et al. (39) used SELDI-TOF MS (surface-enhanced laser desorption/ionization time-of-flight mass spectrometry) for finding proteins that are significantly elevated in RA patients. Interestingly, along with SAA of 11,682 Da, two truncated and probably post-translationally modified SAA proteins were also identified: SAA without its first N-terminal Arg residue of 11,526 Da (SAA des-Arg) and SAA truncated at the N-terminal end by 2 residues, Arg and Ser, of 11,439 Da (SAA des-Arg/des-Ser). Li et al. (40) confirmed the differential expression of SAA in the serum of RA patients compared to healthy controls by proteomics. Seok et al. (41) used a nanoliquid chromatography-tandem mass spectrometry (LC-MS/MS) to identify candidate biomarkers for RA pre-screening. They found the concentration of SAA4 in the serum of clinically healthy individuals with high rheumatoid factor (RF) values significantly higher compared to sera with normal RF values. Furthermore, by using ELISA, they validated a significantly up-regulated SAA4 in RA patients. Therefore, SAA4 may represent a novel prescreening marker for early RA detection. Remarkably, SAA4 was found to be superior to CRP as RA biomarker, and the combination of SAA4 and CRP had even higher pre-screening efficacy. Nys et al. (42) investigated SAA1 and SAA2 isoforms and their allelic variants in patients with early-onset, weak/moderate and severe RA, AS, SLE, OA and healthy controls. They found SAA2 levels significantly higher in all the RA patients compared to controls and other pathologies (SLE; OA), while SAA1β levels were increased only in early-onset RA and SAA1α in severe RA. The weights of SAA1α and SAA1β levels in the total SAA response were different according to the studied pathologies and RA severity. In particular, SAA1α made up to 80% of total SAA in RA while SAA1β made up to 69% in SLE. This leads to the conclusion that SAA2 and SAA1β isoforms can serve as RA biomarkers before the symptoms onset (along with RF and anti-CCP), while the SAA1α/SAA1β ratio is useful for evaluating disease severity and response to treatment. Furthermore, this study demonstrated that SAA is not only quantitatively different among various inflammatory pathologies, but also qualitatively by different representation of isoforms.

In conclusion, described observations (as summarized in **Table 1**) indicate that assessment of the commonly used markers of disease activity (CRP or ESR) is insufficient for evaluation of the disease activity in RA. Moreover, persistent elevated SAA levels represent subclinical inflammation and a risk for developing amyloidosis. Subsequent determinations of SAA serum levels could therefore be useful for recognizing patients in a need of a more intensive treatment with biologic immunotherapy. Additionally, the identification of various SAA truncated isoforms by proteomics, which is not possible by ELISA, may be of importance because of their possibly different pathophysiological roles. For the time being, only SAA forms with a role for diagnosis have been investigated, while further studies should explore the specificity of these SAA forms for different rheumatic diseases and their value in monitoring disease activity and predicting disease course.

## SAA in Juvenile Idiopathic Arthritis

Filipowicz-Sosnowska et al. (43) and Scheinberg et al. (44) were the first to report a significant correlation between SAA concentration and disease activity in JIA patients. Moreover, both groups noticed significantly higher levels of SAA in systemic and polyarticular forms of JIA than in the oligoarticular type. Intriguingly, the first two forms have higher incidence of amyloidosis. However, in both studies, SAA levels were not different between JIA patients with secondary amyloidosis and those without, indicating that SAA levels cannot be used for detecting amyloid deposits in JIA patients. Kutulculer et al., Cantarini et al. and Dev et al. (45–47) discerned SAA as a more sensitive marker than CRP or ESR for assessing JIA disease activity defied by the presence and number of active joints assessed by clinical and ultrasonographic examination. In addition, SAA was elevated in 18% of patients with normal ESR and 28% of patients with normal CRP (47). This suggests that SAA should be used as a marker of disease activity in JIA patients, especially in terms of low disease activity.

Miyamae et al. (48) explored differentially expressed proteins in systemic JIA patients. Paired sera from each patient were analyzed prior to and after the treatment with conventional or biologicDMARDs, using the SELDI-TOF MS proteomic analysis. Despite the small number of patients (n=23), highly significant and consistent changes were observed, with SAA (11.6 kDa) showing the biggest decrease in expression upon the treatment. In addition, responders and non-responders to conventional therapy had significant differences in baseline SAA expression, suggesting clinical utility of SAA for both predicting and monitoring response to therapy in JIA patients. Articles concerning utility of SAA in JIA patients are summarized in **Table 2**.

## SAA in Ankylosing Spondylitis

Many studies have shown that SAA can be used as a marker of disease activity and response to therapy in AS patients (**Table 3**). Lange et al. (49) evaluated SAA as a marker of disease activity in AS and found a significant correlation with Bath Ankylosing Spondylitis Disease Activity Index (BASDAI), the established disease activity score. Moreover, Jung et al. (50) reported that SAA is superior to CRP and ESR in the detection of AS inflammatory activity. SAA and BASDAI score were elevated and positively correlated even in AS patients with normal ESR (42% of all patients) and CRP levels (24% of all patients included in the study).

De Vries et al. investigated the usefulness of SAA for predicting and monitoring response to anti-TNFα therapy (infliximab or etanercept) in AS patients (51). They found that normal baseline levels of both CRP and SAA were significantly associated with nonresponse to therapy, while elevated baselines of each of these acute phase proteins had a high predictive value for response. The combination of elevated baseline CRP and SAA levels was the strongest predictor of response to anti-TNFα therapy, suggesting these baseline values should be added to anti-TNFα response criteria in order to facilitate selection of AS patients who are likely to respond to this kind of treatment. Interestingly, a secondary increase of SAA levels after initial normalization was associated with developing antibodies against infliximab. Therefore, monitoring SAA levels might be used for detecting anti-drug antibodies even before the loss of response to adalimumab and clinical deterioration. This intriguing finding was recently verified in a large cohort of IBD patients (n=805) receiving adalimumab by Rubin et al. (189). SAA concentrations were significantly decreased after introducing adalimumab to therapy. After initial normalization, SAA levels significantly increased in patients who developed anti-adalimumab antibodies. High titter of these antibodies were associated with 4.8-fold increase in the SAA expression.

Li et al. (52) confirmed SAA as a diagnostic indicator of AS by a proteomic approach (MALDI-TOF MS). Rademacher et al. (53) validated increased SAA levels in AS patients but did not found a significant correlation between baseline SAA levels and radiographic spinal progression after two years. However, baseline SAA was measured in a cohort of patients with advanced AS (mean disease duration was 15 years). Therefore, before reaching the final conclusion, the further studies evaluating SAA as a predictor of the radiographic progression in early-stage AS patients is needed.

Recently, Wu et al. (54) discovered 7 over-expressed proteins in the sera from AS patients compared to healthy controls by using an human antibody array. In this study, a protein with the highest differential expression was SAA1, even in patients receiving adalimumab. Moreover, SAA1 was significantly higher in patients with peripheral joint involvement and significantly decreased after 24-weaks of adalimumab therapy. Liu et al. (55) also performed a proteomic analysis of AS patient’s sera and found that the combination of CRP and SAA1 has the highest sensitivity and specificity for AS diagnosis and disease activity. They confirmed that SAA1 is more sensitive than total SAA in differentiating active from stable AS as well as AS from healthy controls. All participants in this study were treatment naïve and without comorbidities, making the results highly reliable and worth of further exploration.

## SAA in Different Types of Vasculitis

As in other IRD, studies have shown that SAA is a potentially good biomarker of diseases activity and response to therapy in patients with Takayasu arteritis (TA) and other types of vasculitis (**Table 4**). Ma et al. (56) and Nair et al. (57) found circulating SAA levels significantly higher in TA patients with active disease compared to those with inactive disease. Nevertheless, SAA levels in the inactive group were still higher than in healthy controls, suggesting an ongoing subclinical inflammation, which was occasionally confirmed by a FDG PET-CT (fluorodeoxiglucose positron-emission computed tomography) and led to therapy adjustment. In the same manner, no significant differences were found for CRP. Furthermore, Nair et al. (57) analyzed changes in biomarker levels and disease activity as a response to therapy (mycophenolate mofetil, azathioprine or methotrexate). SAA levels significantly decreased during the follow-up of mean duration of 7.5 months in treatment responders, while there were no significant changes in non-responders. The relative changes in SAA values during follow-up reflected the response to treatment more accurately than the same changes in CRP or ESR values. These results are limited by a small cohort size, hence further studies with a longer follow-up and in a larger cohort should ascertain the utility of monitoring SAA levels in the management of TA patients.

Lately, an important role of SAA in the pathogenesis of giant cell arteritis (GCA) has been recognized. Along with proangiogenic properties and the induction of cell growth and angiogenesis mediated by TLR2, O’Neil et al. (190) proved that SAA is directly secreted in inflammatory temporal arteries. Hocevar et al. (58) reported a predictive value of high baseline SAA levels (measured at diagnosis) for an early relapse in patients with GCA receiving corticosteroids, indicating a clinical utility of SAA in an early identification of non-responders to corticosteroid therapy. The correlation of baseline SAA with relapse was more significant than that of CRP or ESR. The reported correlations are limited by a small cohort size and potential selection bias since the study was performed at one department in a single centre. Burja et al. (59) identified SAA as the most differentially expressed serum biomarker between patients with GCA and HC (83-fold increase in patients) out of 48 tested laboratory parameters (including CRP and ESR). All patients included in the study were in early disease stage, treatment naïve and were followed up for at least 1 year. Changes in SAA levels were better correlated with disease activity than changes in CRP or ESR levels. Considering the large proportion of smokers (38%), type II diabetes (14%) and hypertensive patients (53%) in the study cohort, as well as the fact that healthy controls significantly differed in age (median age of 74.1 in patients vs. 50.8 in healthy controls), future research should corroborate the observed correlations. Dartevel et al. (60) confirmed clinical utility of SAA in distinguishing GCA patients with active from those with inactive disease. Despite tendency to higher SAA concentrations in microbial infections, they observed no statistically significant difference between active disease and infection, similarly to Van Sleen et al. (61). Intriguingly, SAA was significantly correlated with serum IL-6 levels only in GCA but not in infection group, suggesting pathophysiological differences. The latter group also confirmed SAA expression at the tissue level (temporal artery biopsy), emphasizing its important role in GCA.

The clinical applicability of SAA has also been investigated in Kawasaki disease (KD). Mitani et al. (62) revealed a significant association of elevated SAA concentration and persistence of coronary artery lesions (aneurysms, stenosis or occlusion) late after KD (mean time after onset was 10 years). This association was supported by logistic regression analysis (adjusting for age, smoking, BMI, blood pressure, total cholesterol/HDL ratio) and was stronger for SAA than for CRP. When interpreting the results, a caution is needed since the study was cross-sectional and the number of patients was relatively small, so large cohort corroboration is required. Whitin et al. (63) published an intriguing report on a novel truncated SAA form in patients in Kawasaki disease. By using a proteomic approach (SELDI-TOF MS), the group investigated differences in serum protein expression between children with KD and febrile children with at least 3 day-long fever and at least one of the clinical criteria for KD. One mass spectrometry peak (7,860 Da) had significantly higher intensity in children with KD than controls, along with a significant difference among acute and subacute KD patients. Noteworthy, the peak, which was identified as truncated form of SAA with N-terminal at Lys-34, disappeared when the symptoms resolved. Moreover, the possibility of ex vivo SAA proteolysis was excluded by the presence of the truncated form even when the blood samples were collected in tubes containing protease inhibitors. Nevertheless, the relevance of this SAA form as a diagnostic biomarker in KD and possibly in other types of vasculitis as well as its place in pathogenesis is yet to be discovered. Moreover, it is important to underline that for the peptide identity confirmation the hybrid mass spectrometry immunoassay technique was used, since a conventional ELISA would not have detected the truncated SAA peptide because its signal would have been a minor contributor to the overall plasma SAA content.

Finally, Purevdorj et al. (64) identified SAA as the most sensitive biomarker for diagnosis of Henoch-Schonlein purpura (HSP) among 12 tested biomarkers (including CRP), however no significant difference was found between SAA levels in HSP and sepsis. In a cross-sectional study by Kuret et al. (65). SAA levels were significantly increased (12-fold) in the sera of adult patients with IgA vasculitis compared to healthy controls. Based on these reports, the potential clinical utility of SAA in IgA vasculitis in both children and adults is worth of further studies.

## SAA in Sarcoidosis

SAA has been evaluated as a marker of sarcoidosis in many reports (summarized in **Table 5**). Chen et al. (191) demonstrated a more intense SAA expression in the sarcoidosis granuloma compared to other granulomatous diseases, suggesting it as a diagnostic biomarker for sarcoidosis. SAA also emerged as a key protein regulating granulomatous inflammation through TLR-2. Serum levels of SAA are increased in all sarcoidosis patients but reports regarding SAA correlation with sarcoidosis severity are non-consistent. Rothenkrantz-Kos et al. (66) defined disease severity by chest radiographs and lung function test results and found no correlation with SAA levels. They characterized SAA as a sensitive (96%), but not specific (37%) diagnostic marker for sarcoidosis (cut-off level of 2.5 mg/L). However, the control samples in this cross-sectional study came from a cohort of a not well-defined ostensibly healthy donors and the treatment administered to patients was not reported. In a study by Miyoshi et al. (67) baseline SAA levels were not predictive for an increased lung infiltration in patients with sarcoidosis, although SAA was not influenced by immunosuppressive therapy unlike the commonly used ACE (angiotensine-coverting enzyme). On the other hand, Salazar et al., Bargagli et al. and Gungor et al. (68–70) reported significantly higher SAA levels in patients with active sarcodiosis than in patients with inactive disease. Moreover, Bargagli et al. (69) found a predictive value of SAA for prolonged steroid requirement. Interestingly, proteomic analysis revealed two highly expressed SAA1 isoforms in all of the sarcoidosis sera and in none of the sera from healthy controls. These SAA1 isoforms could match the unidentified biomarker of sarcoidosis previously reported in a proteomic study by Bons et al. (192). The latter group used SELDI-TOF MS and reported two unidentified serum proteins that were up-regulated in the sarcoidosis sera. The molecular weights of those proteins (11,995 and 11,734 Da) correspond to those of the two SAA1 isoforms in the study by Bargagli et al. (69). and Bons et al. reported the high sensitivity and specificity of these proteins for sarcoidosis (192). Zhang et al. (71) confirmed that SAA was significantly higher in the sera from sarcoidosis patients compared to patients with other pulmonary diseases including tuberculosis (the sensitivity of 96.3% and specificity of 52.3% at the cutoff value of 101.98 mg/L). Furthermore, by a proteomic analysis, a unique protein peak of 3210 Da with the highest expression in sarcoidosis sera was revealed. The peak was identified as the N-terminal peptide of 29 amino acids of SAA. Additionally, immunohistochemical staining showed more intense SAA depositions in lung tissue in sarcoidosis than in other groups, suggesting SAA to be used in differential diagnosis of sarcoidosis from other pulmonary diseases. Contrarily, Enyedi et al. (72) reported no differences in SAA or CRP levels between patients with biopsy-proven sarcoidosis and biopsy negative patients. Nevertheless, it must be noticed that the latter group had histological diagnoses of lymphoma, carcinoma, histiocytosis, anthracosis, etc, all of which are associated with increased markers of inflammation.

In light of these findings, the utilization of SAA1 as well as truncated SAA forms as markers for diagnosis, assessing disease activity and response to therapy in patients with sarcoidosis requires additional research.

## SAA in Other Inflammatory Rheumatic Diseases

SAA has been investigated in other rheumatic diseases as well, as shown in **Table 6**.

In patients with systemic sclerosis (SSc), SAA levels are elevated and correlate with disease severity. Brandwein et al. (73) were first to investigate SAA as a marker of disease activity in SSc. The group reported elevated SAA concentrations in 98% of their patients and a significant correlation with disease severity (determined by extension of skin thickening). Lakota et al. (74) included patients with limited and diffuse SSc in the study and found elevated SAA levels in 25% of patients, while Lis Swiety et al. (75) included only patients with diffuse SSc and detected increased SAA levels in 66%. The discrepancy between these results may be explained by inclusion or exclusion of limited SSc patients and different SAA cut-off values between the studies. Furthermore, both of these studies found a significant correlation between SAA levels and deterioration of lung function (assessed by forced vital capacity, diffusion capacity for carbon monoxide or reticulation pattern on chest CT). According to these studies, SAA is superior to CRP as a marker of pulmonary involvement in SSc. Still, longitudinal studies are needed to validate SAA as a marker of disease activity, predictor of disease progression and response to therapy in SSc.

The only article reporting on SAA in SLE patients that was found through comprehensive literature search is the study by Wang et al. (76). The group revealed a significant correlation between SAA levels and SLE disease activity (determined by SLE disease activity score—SLEDAI). Although SAA was significantly correlated with serum levels of hs-CRP (high sensitivity CRP) and ESR, a binary logistic regression analysis showed that SAA values are independently associated with active SLE. Since this research was retrospective and cross-sectional, a prospective, longitudinal, large cohort study is necessary to confirm the clinical utility of SAA for monitoring SLE patients.

Boyd et al. (77) reported that among 15 biomarkers (including CRP), SAA levels were most significantly correlated with disease activity in patients with psoriatic arthritis (PsA). However, the study was cross sectional in a small cohort (45 patients) and included patients receiving different therapies (including conventional and biological DMARDs). Moreover, disease activity assessment did not include ankle and feet involvement that are frequently seen in PsA. Future studies should therefore validate the utility of SAA in monitoring PsA patients.

## SAA in Systemic Autoinflammatory Diseases

Amongst the systemic autoinflammatory diseases (SAID), the clinical utility of SAA has been most extensively investigated in patients with FMF. SAA levels are elevated in FMF patients not only during attacks, but also in the attack-free period revealing a sustained subclinical inflammation. Furthermore, asymptomatic MEFV mutation carriers also have an increased SAA concentration potentially leading to the development of amyloidosis.

Duzova et al. (78) found SAA levels above the reference range in more than 95% of the FMF children in between the attacks, even though 50% of them had not experienced attacks within the last 12 months. SAA was shown to be the best biomarker of subclinical inflammation in FMF (compared to CRP, ESR, ferritin and fibrinogen). An increase in the colchicine dose resulted in a dramatic decrease of SAA concentration advocating the use of SAA for therapy guidance. Lachmann et al. (79) reported significantly elevated SAA levels (>3 mg/L) in MEFV mutation carriers and in the attack-free period in more than 70% of their patients, even though all patients were under colchicine therapy. Moreover, a remarkable degree of acute-phase activity (measured monthly by SAA and CRP levels) led the authors to question about the participants compliance. This suspicion was eventually confirmed in a considerable number of the patients. Therefore, they suggested to measure frequently SAA in patients with FMF for reinforcing their therapy compliance. Berkun et al. (80) confirmed elevated SAA levels in the attack-free period and in MEFV mutation carriers. However, in this report SAA was increased (>6 mg/L) in only 25% of FMF patients between attacks compared to 70% reported by Lachmann et al. (79). The discrepancy between the results may be due to the different definition of elevated SAA levels as well as the difference in therapy doses and perhaps compliance to colchicine therapy. In 30% of patients SAA measurement led to a change in colchicine dose and, consequently, SAA level normalization. Another interesting finding was significantly higher SAA in noncompliant patients than in nonresponders to therapy, therefore supporting suggestion of Lachmann et al. for frequent SAA measuring for distinguishing these two groups and reinforcing compliance.

Yalcinkaya et al. (81) validated SAA level above reference range in FMF patients during the attack and the attack-free period in the same patients. Similarly, increased SAA levels were observed in clinically healthy FMF heterozygotes. In patients that suffered from chronic inflammatory diseases or chronic infections with a high potential for developing secondary amyloidosis, SAA concentrations were not higher than in those with acute infections with an almost zero chance for developing amyloidosis, implying SAA has no predictive value for amyloid formation. Another important observation was lack of significant difference between SAA levels in children with FMF exacerbation and those with acute infections, although in both groups SAA was significantly increased. Nevertheless, it must be noticed that all FMF patients were receiving colchicine at the time of the study, which is known for reducing SAA levels. Kallinich et al. (82) validated elevated SAA levels in FMF patients at diagnosis, during attacks, in between the attacks and in MEFV mutation carriers. Lofty et al. (83) found increased SAA (>30 mg/L) in 79% of FMF patients two weeks after the last attack. Only 31% had elevated CRP concentration, indicating that SAA can persist elevated after FMF attacks more than CRP. More recently, Cakan et al. (84) reported that SAA is significantly higher in children with acute FMF attacks than in children with acute febrile respiratory infection and therefore can be used for differentiating those two clinical entities. Again, of note is that all FMF patients were treated with colchicine. At the cut-off value of 111.5 mg/L, the SAA sensitivity for discriminating FMF attack from acute infection was 100%, and the specificity was 65.1%. Since these results are opposite to those of Yalcinkaya et al. (81), further research with a larger sample size and before introducing colchicine to therapy are necessary to determine whether SAA provides additional value compared to CRP in suspected acute FMF attacks.

Since SAA is considered to be involved in the pathogenesis of atherosclerosis, some of the research investigated a potential value of SAA for predicting atherosclerosis in patients with FMF. Bilinger et al. (85) and Mohamed et al. (86) found a significant correlation between SAA levels and intima media thickness of the common carotid artery (CIMT)—an early marker of atherosclerosis. The patients studied were receiving NSAIDS and colchicine, so SAA levels were suppressed, but still higher than normal. Possibly an even stronger correlation would have been found if the patients were untreated. Sargsyan (87) found SAA significantly higher in FMF patients with any kind of vascular involvement than in those without. The role of SAA in atherosclerosis needs to be further explored, on both molecular and clinical level.

Taken all together, measurement of SAA in FMF patients should be used in evaluating disease activity, risk of amyloidosis and atherosclerosis, as well as response to therapy. Moreover, determination of SAA level may serve as a screening test for asymptomatic family members to determine the need for genotyping. However, further studies are required to determine the clinical benefits of SAA normalization by increasing colchicine dose in the asymptomatic patients. Furthermore, the target SAA level for colchicine dose modification should be defined as well as the needed frequency and time of longitudinal monitoring of SAA levels in the asymptomatic FMF individuals before making adjustments.

Except in FMF, benefits of SAA level monitoring have been explored in other SAIDs. In Behçet's disease (BD), a multifactorial SAID, SAA might not be useful for assessing disease activity, but is positively correlated with major organ involvement and can be used for identifying patients at higher risk of life-threatening complications. Aygunduz et al. (88) reported SAA as a more sensitive and specific marker for BD than CRP, advocating the use of SAA as diagnostic marker and indicator of subclinical inflammation in BD. Contrarily, Cantarini et al. (89) and Vitale et al. (90) reported no significant difference of SAA levels between patients with active and inactive BD (assessed by Behcets disease current activity form—BDCAF), but they found SAA levels associated with skin involvement. Vitale et al. (90) suggested SAA as an indicator of oral aphthosis, neurological and ocular involvement in BD because of the strong correlation between these factors (SAA serum levels higher than 30, 50, and 150 mg/L, respectively). Interestingly, SAA was found to be significantly correlated with homocysteine serum levels (indicator of vascular involvement). This possible use of SAA for indicating vascular involvement and predicting thrombotic risk in BD patients should be of interest for future studies. Recently, Sota et al. (91) confirmed no association between SAA levels and BD activity (BDCAF) but found a significant association between SAA levels above 200 mg /L and a major organ involvement as well as between SAA levels above 150 mg/L and ocular, skin or mucosal manifestations. They suggested SAA as a predictor of major organ involvement and ocular disease relapse in BD. Lee et al. (92) validated a non-significant correlation between SAA and disease activity in BD patients by proteomic analysis. However, this study included BD patients with only intestinal involvement, so future studies should explore biomarkers of BD patients with multisystemic involvement by a proteomic approach. All of the abovementioned studies included a small number of patients, so large cohort studies are needed to confirm these potential advantages of monitoring SAA in patients with BD.

Monitoring SAA levels has also been incorporated in evaluating patients with Muckle-Wells syndrome (MWS). Hawkins et al. (93) and Scarpioni et al. (94) reported that SAA serum levels and clinical symptoms are concomitantly significantly increased after introducing biologic therapy in MWS patients (anakinra and canakinumab, respectively). Hoffman and co-workers (95) used SAA together with hs-CRP as serum biomarkers of efficacy of rilonacept in CAPS patients, as well as previously reported study by Goldbach-Mansky et al. (96). The latter study found that the change in SAA level as a response to rilonacept therapy is better correlated with improvement in clinical symptoms than CRP or ESR levels. In cases of reoccurrence of disease flare, SAA levels significantly increased despite the rilonacept treatment. Wiken and co-workers (97) confirmed utility of SAA as a marker of response to anakinra in MWS and neonatal onset multisystem inflammatory disease (NOMID) patients, as well as a high incidence of anti-drug antibodies development, but with no influence on efficacy of anakinra or SAA levels. These findings highlight the potential utility of SAA in assessing response to biologic therapy in MWS and NOMID patients. The articles discussed in this section are summarized in **Table 7**.

## SAA in Secondary (AA) Amyloidosis

The association between SAA and secondary (AA) amyloidosis was one of the first investigated roles of SAA, with many new studies still emerging (**Table 8**). Amyloidosis is a single- or multiorgan disease characterized by extracellular tissue deposition of low-molecular weight, insoluble and amorphous proteinaceous material, causing progressive organ dysfunction. Rheumatic and autoinflammatory diseases are associated with a high rate of secondary (AA) amyloidosis. As already mentioned, SAA gene polymorphisms have an influence on developing amyloidosis. The strong correlation between amyloid deposition and SAA1γ allele in Japanese RA patients was confirmed by Ishii et al. (99). Furthermore, the group reported significantly higher SAA levels in RA patients with amyloidosis than in those without. SAA showed a stronger correlation with the presence of amyloid deposits than CRP. However, according to the most of published reports, SAA serum levels are not correlated with the presence of amyloid deposits (43, 44, 100, 101). Therefore, contrary to expectations, high SAA levels are a prerequisite, but not a sufficient condition for developing amyloidosis and cannot be used as a diagnostic marker for amyloidosis. It is possible that increased proteolytic cleavage of SAA contributes to the development of amyloidosis. Indeed, Migita et al. (100) detected, in addition to the full-length SAA protein, 6 kDA and 4.5 kDa SAA-derived fragments in the sera of RA patients. The ratio of these fragments to total SAA was significantly higher in patients with than in those without amyloidosis, confirming the increased proteolysis hypothesis. The potential use of these truncated SAA forms as diagnostic markers for amyloidosis should be further investigated.

Subsequent studies in amyloidosis patients revealed some clinically important properties of SAA. Although SAA may not be a diagnostic or predictive marker of amyoidosis, once amyloidosis has developed, the SAA levels over the course of the disease represent the main factor affecting amyloidosis progression and survival. The kidney is one of the most frequent sites of amyloid deposition and, without treatment, amyloidosis-associated kidney disease usually progresses to end-stage renal disease (ESRD). Prognosis of renal amyloidosis significantly correlates with the SAA concentration. Falck et al. (6) and Lachmann et al. (102) reported a strong correlation between the mean SAA value and changes in renal function in patients with renal amyloidosis, suggesting SAA for predicting renal deterioration. Reportedly, renal improvement is expected when SAA median is less than 6 mg/ml and deterioration when SAA median is above 28 mg/L.

Furthermore, SAA levels are significantly connected to changes in amyloid load and long-term survival in amyloidosis. In a study by Gilmore et al. (103), SAA values were significantly higher among patients with further amyloid accumulation than in those with stable amyloid load in whom SAA levels were still higher than in patients with deposit regression. According to a large cohort prospective study of amyloidosis by Lachmann et al. (102), patients with SAA concentrations in the low-normal range (<4 mg/L) have relatively favorable outcome, while persistent elevation of SAA is a powerful risk factor for progression to ESRD and death. The risk of death is 17.7 times higher among patients with uppermost SAA concentrations (>155 mg/L) than in those with SAA concentrations below 4 mg/L. Even in the patients with slightly elevated SAA concentrations during follow-up (4–9 mg/L), the risk of death is 4 times increased. Interestingly, decrease in median SAA level below 10 mg/L is associated with the regression of amyloid deposits. Therefore, therapy that decreases SAA production to within the reference range prevents further accumulation of amyloid deposits and can stabilize or even reverse existing amyloid deposits leading to a better long-term survival. Based on these findings, frequent SAA measurements in patients with secondary amyloidosis should be used for therapy guidance. However, median SAA concentration and status of amyloid deposits varied substantially between individuals in these studies, possibly due to the differences in underlying diseases and anti-inflammatory treatment or comorbidities (obesity, diabetes, hypertension, tobacco consumption) that may influence SAA levels.

Finally, SAA can be used in monitoring response to biologic therapy in rheumatic patients with AA amyloidosis. A number of recent studies have verified that therapeutic strategies involving IL-6 inhibitors and TNFα inhibitors result in a decrease of serum SAA level and consequently represent an excellent therapeutic strategy for AA amyloidosis. Perry et al. (104) and Nakamura et al. (105) verified concomitant decrease in SAA concentration, disease activity and proteinuria in RA patients with AA amyloidosis receiving etanercept (anti-TNFα antibody). Miyagawa et al. (106) and Lane et al. (107) studied the efficacy of tocilizumab (TCZ, anti-IL-6 receptor antibody) in patients with AA amyloidosis. In all patients, SAA levels significantly decreased together with a remarkable decrease in proteinuria, regression of amyloid deposits and significant improvement in clinical symptoms. Okuda et al. (108) compared the effects of anti-TNFα (etanercept, infliximab and adalimumab) and anti-IL-6 (TCZ) therapy against AA amyloidosis by measuring SAA levels. Along with a more imposing improvement in eGFR and amyloid regression, SAA concentration decreased more significantly in the anti-IL-6 group than anti-TNFα group. Taken all together, SAA seems to be a reliable marker of response to biologic therapy in rheumatic patients with secondary amyloidosis.

## SAA in COVID-19

Since we are currently experiencing a pandemic of COVID-19, we summarized 6 articles reporting on SAA as a biomarker in patients with COVID-19 found in our research through PubMed and Scopus databases (**Table 9**). All of the articles were published in 2020.

In critically ill COVID-19 patients a cytokine storm with highly elevated IL-6 has been described (193). Since SAA is correlated with IL-6 and involved in the pathogenesis of the risk conditions for severe COVID-19 (obesity, diabetes and atherogenesis), it might also play a role in the pathogenesis of COVID-19 and therefore present a potential biomarker and therapeutic target.

SAA is increased in all COVID-19 patients with the mean SAA value 4 times higher in critically ill than in mild-ill patients (109). Moreover, Shi et al. (110) reported SAA levels were increased whereas CRP levels were normal in more than 20% of patients. While in patients with both SAA and CRP within the reference range no severe pneumonia occurred, in some of the patients with normal CRP but elevated SAA severe pneumonia was found, suggesting higher sensitivity of SAA for assessing COVID-19 severity. Wang and co-workers (111) validated more significant correlation between disease severity and SAA level than CRP or ESR. In addition, they found SAA value above 100 mg/L as an indicator of disease progress to the critical stage. Huan Li et al. (112) reported SAA and SAA to lymphocyte count ratio as sensitive indicators of COVID-19 severity and prognosis (more sensitive than CRP or PCT). The initial SAA level was correlated with future dynamic changes of CT scans meaning that patients with higher initial SAA levels are more likely to have worsening of CT scans. Interestingly, initial SAA was found to have a higher predictive value for disease progression than the initial CT scan. A retrospective study of COVID-19 death cases revealed that, among included serum biomarkers, only SAA was significantly elevated in all of the patients with fatal outcome (113). Mo et al. (114) showed by logistic regression analysis that SAA, but not CRP, can serve as an independent predictive factor of COVID-19 course. At the cut-off value of 122.9 mg/L, SAA can predict acute exacerbation with an accuracy of 89.1%.

To conclude, SAA might give additional information about COVID-19 severity and prognosis to more commonly used biomarkers. Therefore, SAA measurement should be included in managing COVID-19 patients. At the present moment, to our knowledge, there are no published data on SAA as a biomarker of COVID-19 severity in patients with IRD, so future studies should explore the clinical relevance of SAA as a biomarker in the new era of coexistence of IRD patients and SARS-CoV-2.

## Limitations of SAA as a Biomarker of Inflammatory Rheumatic Diseases 

Despite many discussed advantages provided by the use of SAA as a biomarker in various rheumatic and autoinflammatory diseases, as with many other biomarkers, there are several limitations arising mostly from the different protein isoforms, genotype and measurement methods.

Firstly, most of the studies that investigated the role of SAA in disease pathogenesis used recombinant human SAA (rhSAA) that differs from the native SAA1 and SAA2 isoforms by 2 amino acids, resulting in a hybrid of SAA1 and SAA2. Some researchers reported differences between rhSAA and endogenous SAA proinflammatory functions (194, 195), thus further experiments should use isolated endogenous SAA from synovial fluid or explants models to identify the effects of SAA in the *in vivo* environment.

Although SAA has been described as the most suitable inflammatory marker for certain rheumatic diseases more than five decades ago, it is still not used as a common biomarker of disease activity in clinical practice. CRP has been widely used as a routine clinical test, while SAA is less popular mostly due to technical difficulties in large scale purification of SAA, stable production of antibodies with high titer, development of an assay system, and standardization of the assay. Various assay methods for SAA quantification have been used. Radioimmunoassay (RIA), radial immunodiffusion and enzyme-linked immunosorbent assay (ELISA) are highly sensitive (detection limit 0.2 μg/L) but time-consuming and therefore inconvenient for clinical use. On the other hand, immunonephelometric and immunoturbidimetric assay are rapid and fully automatic, but have relatively low sensitivity (detection limit >3mg/L). In addition, the commercially available kits for SAA are based on polyclonal antibodies which lack isotype specificity.

Furthermore, SAA genotype may also influence baseline SAA levels (99, 196–198), indicating the need for an individual approach when using SAA plasma levels for assessing disease activity. Unsurprisingly, there are significant variations in the absolute values of SAA among research groups. Even physiological SAA concentrations vary substantially among studies (0.1–10 mg/L) what might have led to critical errors because in some cases even 100-fold increase could not be detected. Additionally, comorbidities such as epilepsy, diabetes and other chronic inflammatory diseases, as well as drugs such as statins and dietary supplements including vitamins A and E and polyunsaturated fatty acids can influence SAA plasma levels along with alcohol use, smoking and obesity (199–203). Obese but otherwise healthy patients have elevated SAA plasma levels and diet-induced weight loss is associated with significant decrease in plasma SAA level. This effect is proportional to the amount of weight lost but independent of dietary macronutrient composition (199). Ethanol induces the SAA production in a dose-dependent manner (202). Tobacco smoking significantly increases serum SAA level and the increase is correlated to the degree of smoking (203). Therefore, all of these variables should be taken in consideration when analysing research results.

As a consequence of these limitations, reliable testing and laboratories that measure SAA levels are not widely available, and data about levels expected in diseases are limited. In time, the availability of assay methods and their wider use should corroborate variables that have a significant impact on SAA levels and provide a clearer picture when interpreting results.

## Conclusion

Although SAA was sporadically used as a biomarker in many chronic diseases for the past five decades, the use of other inflammatory biomarkers, such as CRP and ESR, has overwhelmed its use in clinical practice. Advantages of these commonly used biomarkers for being cost-effective and easily applicable are also associated with limitations of sensitivity and specificity especially in settings of low-activity rheumatic disorders. In the era of biological therapy, the need for a new biomarker for predicting disease activity and monitoring remission and relapse for various rheumatic diseases has been emphasized. With the discovery of new disease mechanisms and development of proteomic techniques as the most effective methods for identifying molecular markers of disease activity and treatment response, SAA started to regain its importance. In such circumstances, we aimed to collect and summarize all the relevant articles on the clinical utility of SAA in a number of rheumatic and systemic autoinflammatory diseases.

Although articles included in this review are very heterogeneous in design, subjects, parameters measured and results, the general conclusion is that SAA plays an important role in the pathogenesis and clinical course of rheumatic diseases. SAA is involved in many processes important for initiation, perpetuation and resolution of chronic inflammation in IRD. Furthermore, SAA is a sensitive biomarker of disease activity and indicator of the disease prognosis and therapeutic response in a wide range of immune mediated IRD (**Table 10**). In plenty of studies SAA has been demonstrated to provide more information and higher sensitivity than CRP, especially in a state of subclinical inflammation, as well as in patients receiving glucocorticoids or conventional or biologic immunosuppressive therapy. According to the results of proteomic analyses, specific SAA forms have even higher sensitivity and specificity for certain diseases than the total serum SAA value.

**Table 10:** Summary of possible uses of SAA concentration monitoring in different chronic inflammatory diseases and relevant studies.

Disease	Possible uses of SAA concentration follow-up	Relevant studies, reference number
Rheumatoid arthritis	Diagnostic marker;Biomarker of disease activity;Indicator of subclinical inflammation;Predictor of clinical outcome;Indicator of therapeutic response;Indicator of risk for amyloidosis;Indicator of cardiovascular risk;	(9–42)
Juvenile idiopathic arthritis	Biomarker of disease activity,Indicator of subclinical inflammation;Indicator of risk of developing amyloidosis;Indicator of therapeutic response;Predictor of response to therapy;	(43–48)
Ankylosing spondylitis	Biomarker of disease activity:Indicator of subclinical inflammation;Indicator of therapeutic response;Predictor of response to therapy;	(49–55)
Takayasu`s arteritis	Biomarker of disease activity;Indicator of subclinical inflammation; Indicator of therapeutic response;	(56, 57)
Giant cell arteritis	Biomarker of disease activity;Predictor of response to therapy;	(190–61)
Kawasaki disease	Indicator of persistence of coronary artery lesions;	(62, 63)
IgA vasculitis	Diagnostic marker;	(64, 65)
Sarcoidosis	Diagnostic marker;Biomarker of disease activity;Predictor of future prolonged steroid requirement;	(191–72)
Systemic sclerosis	Indicator of pulmonary involvement;	(73–75)
Systemic lupus erythematosus	Biomarker of disease activity;Indicator of cardiovascular risk;	(76, 178)
Psoriatic arthritis	Biomarker of disease activity;	(77)
Familial Mediterranean Fever	Biomarker of disease activity;Indicator of subclinical inflammation;Indicator of need for genetic testing;Indicator of risk for amyloidosis;Indicator of therapeutic response;Guide for therapy adjustment;	(78–86)
Behcet's disease	Predictor of major organ involvement:;Indicator of high risk for life- and sight-threatening complications;	(87–92)
Mucle-Wells syndrome	Biomarker of disease activity;Indicator of therapeutic response;	(93–97)
Amyloidosis	Indicator of renal function improvement/deterioration;Prognostic biomarker;Marker of therapeutic response;	(99–107)

In conclusion, we strongly advocate the use of SAA as a cheap and reliable biomarker for use in everyday clinical practice of a wide range of physicians dealing with rheumatic and other immune mediated inflammatory diseases in both children and adults, and propose exploration of clinical utility of specific SAA isoforms in future studies.

## Author Contributions

All authors discussed the contents. IS and LL wrote and edited this manuscript. LL supervised and oversaw the manuscript. All authors contributed to the article and approved the submitted version.

## Conflict of Interest

The authors declare that the research was conducted in the absence of any commercial or financial relationships that could be construed as a potential conflict of interest.
